# Detecting the Molecular System Signatures of Idiopathic Pulmonary Fibrosis through Integrated Genomic Analysis

**DOI:** 10.1038/s41598-017-01765-6

**Published:** 2017-05-08

**Authors:** Indu Gangwar, Nitesh Kumar Sharma, Ganesh Panzade, Supriya Awasthi, Anurag Agrawal, Ravi Shankar

**Affiliations:** 1 Studio of Computational Biology & Bioinformatics, CSIR-IHBT, Palampur, HP India; 2 Centre of Excellence for Translational Research in Asthma & Lung Diseases, CSIR-IGIB, Mall Road, Delhi, India; 3 Academy of Scientific and Innovative Research (AcSIR), Chennai, TN India

## Abstract

Idiopathic Pulmonary Fibrosis (IPF) is an incurable progressive fibrotic disease of the lungs. We currently lack a systematic understanding of IPF biology and a systems approach may offer new therapeutic insights. Here, for the first time, a large volume of high throughput genomics data has been unified to derive the most common molecular signatures of IPF. A set of 39 differentially expressed genes (DEGs) was found critical to distinguish IPF. Using high confidence evidences and experimental data, system level networks for IPF were reconstructed, involving 737 DEGs found common across at least two independent studies. This all provided one of the most comprehensive molecular system views for IPF underlining the regulatory and molecular consequences associated. 56 pathways crosstalks were identified which included critical pathways with specified directionality. The associated steps gained and lost due to crosstalk during IPF were also identified. A serially connected system of five crucial genes was found, potentially controlled by nine miRNAs and eight transcription factors exclusively in IPF when compared to NSIP and Sarcoidosis. Findings from this study have been implemented into a comprehensive molecular and systems database on IPF to facilitate devising diagnostic and therapeutic solutions for this deadly disease.

## Introduction

Idiopathic interstitial pneumonias (IIPs) are interstitial lung diseases with unidentified mechanism, distinguished by matrix deposition and alveolar epithelium disruption. Idiopathic pulmonary fibrosis (IPF) is a chronic fibrosing IIP that has no effective therapy with a mortality rate higher than many cancers; median survival from the time of diagnosis being about three years^1^. There is typically no response to general anti-inflammatory therapy such as glucocorticosteroids, which are effective in some IIPs such as Nonspecific Interstitial Pneumonia (NSIP). Some antifibrotic agents and adenosine receptor antagonist based solutions have shown some limited promise^2–4^. While new targets have emerged, such as proinflammatory cytokines (IL-4, IL-13)^5^, the negligible molecular systems information for IPF remains a problem. A gene expression level understanding of IPF is incomplete, with limited number of studies mostly microarray (MA) based^6–11^ and hardly a couple of RNA-seq studies^12, 13^. Also, very few study has been done covering systems biological perspectives but not specific to IPF^9^. Limited MA based studies with non-coding RNAs have also been performed, but small RNA-seq studies are lacking^14–16^.

From a systems biology perspective, IPF offers both opportunities and challenges. The main challenge is a likely heterogeneity within IPF, which is a clinical diagnosis based on typical diffuse radiological or pathological findings that may be seen in a limited form in other IIPs or even aged lungs. Further, the IPF lung is itself heterogeneous. Thus there is likely to be substantial background noise, as evidenced by high variability between studies. This also presents an opportunity to systematically consolidate all these studies, using computational approach to extract the underlining characteristic signatures for IPF. Such efforts are critical towards better molecular characterization of IPF that could lead to better diagnosis, classification and therapy. This is important because the therapeutic response is very different between different types of IIP and possibly within different subsets of IPF. There is also a need for unified information portal and database for molecular and systems biology of IPF beyond the few existing resources on expression data reporting that have limited scope (https://research.cchmc.org/pbge/lunggens/mainportal.html; http://montgomerylab.stanford.edu/resources.html). The present study has been carried out considering these factors. The study attempts to unify the best available gene expression data for IPF, derive its core characteristics towards generating a computational model of IPF pathology and create a comprehensive state-of-the-art database dedicated to IPF research.

## Results and Discussion

### A set of 39 differentially expressed genes (DEGs) appears critical to determine IPF

While two prior RNA-seq studies (Deng *et al*., Nance *et al*.)^12, 13^ have been conducted, differed in their expression measurement approaches to identify the DEGs and had very limited agreement. After implementing a common expression analysis protocol (Tophat-cuffdiff^17^, see methods), about 70% reads could be mapped, resulting into the identification of 487 and 860 DEGs in the two studies. 124 DEGs were found common between them. However, there was no DEG common across all the gene expression studies in IPF, clearly underlining the poor consensus in this area. This was potentially related to insufficient coverage in some studies due to limited number of MA probes. For example, data from the study done by Yue *et al*.^7^ covered only 4% of the total genes in the human genome.

Because of these irregularities, the two studies performed by Yue *et al*.^7^ and Vuga *et al*.^8^ were discarded. A previous study done by Nance *et al*.^13^ became the starting point for reliable information generation. This RNA-seq study had reported an overlap for 82 DEGs with two MA studies (Meltzer *et al*.^10^, Yang *et al*.^11^) (referred onwards as Set A1). We further found an overlap for 176 DEGs (referred onwards as Set A2) between this RNA-seq and the study done by Cho *et al*.^9^. The intersection of Set A1 and A2 was a set of 39 DEGs (referred onwards as Set A) (Supplementary Figure S1A,B). Since Set A genes were similarly differentially expressed across four different high throughput studies (Cho *et al*., Meltzer *et al*., Yang *et al*. and Nance *et al*.)^9–11, 13^ we expected this set to be a more robust signature of IPF than the previously identified Set A1^13^. To verify this, a comparative k-means cluster analysis was done over the sets while using Z-score transformed expression values.

With Set A1, the clustering analysis achieved accuracy of 92%, 83%, 98%, 100% and 87% (Table 1 Supplementary Figure S2). Clustering based on Set A was better, yielding 92%, 89%, 97%, 100%, and 94% accuracy values (Table 1, Supplementary Figure S3). For the data from an RNA-seq study by Deng *et al*.^12^, the clustering analysis with either set yielded only 67% accuracy. Due to very small size of this set, with three normal and three IPF individuals, this translates to only one misclassification per label. Besides this, these two different sets were also evaluated against the cases of Sarcoidosis and NSIP, the IIP diseases that are part of the differential diagnosis of IPF during clinical evaluation. Both the datasets perfectly distinguished all the cases for Sarcoidosis and NSIP from IPF (Table 1). Set A genes when chosen as the markers for IPF appeared better than previously considered top scoring pair (TSP) approach^9^. Benchmarking of study revealed only 5.1% misclassification of IPF samples with Set A genes while TSP approach had error of 11.2%. Further, TSP appeared biased towards experiments. This suggested potential diagnostic utility for this gene set.

**Table 1:** K-means clustering of Set A1(82 DEGs) and A(39 DEGs) done to distinguish IPF from non IPF samples.

Clustering result with Set A1 genes					
High throughput studies	Total Sample	IPF	non-IPF	Misclassified	Success rate
Meltzer *et al*.^10^	23	17	6	2	92.00%
Cho *et al*.^9^	17	11	6	3	83.00%
Yang *et al*.^11^	169	119	50	5	98.00%
Sanders *et al*.^40^	8	4	4	0	100.00%
Nance *et al*.^13^	15	8	7	2	87.00%
Deng *et al*.^12^	6	3	3	2	67.00%
**Clustering result with Set A genes**					
	**Total Sample**	**IPF**	**non-IPF**	**Misclassified**	**Success rate**
Meltzer *et al*.^10^	23	17	6	2	92.00%
Cho *et al*.^9^	17	11	6	2	89.00%
Yang *et al*.^11^	169	119	50	6	97.50%
Sanders *et al*.^40^	8	4	4	0	100.00%
Nance *et al*.^13^	15	8	7	1	94.00%
Deng *et al*.^12^	6	3	3	2	67.00%
**Clustering result with Set A genes over NSIP and Sarcoidosis samples**					
	**Total Sample**	**IPF**	**NSIP/Sarcoidosis**	**Misclassified**	**Success rate**
Cho *et al*.^9^, Yang *et al*.^43^	23	11	12	0	100.00%
Cho *et al*.^9^, Crouser *et al*.^42^, Lockstone *et al*.^41^	32	11	21	0	100.00%
**Clustering result with Set A1 genes over NSIP and Sarcoidosis samples**					
	**Total Sample**	**IPF**	**NSIP/Sarcoidosis**	**Misclassified**	**Success rate**
Cho *et al*.^9^, Yang *et al*.^43^	23	11	12	0	100.00%
Cho *et al*.^9^, Crouser *et al*.^42^, Lockstone *et al*.^41^	32	11	21	0	100.00%

For each IPF study, samples were clustered under IPF and Non IPF group. Results highlight better performance of Set A genes to classify both type of high throughput studies, contrary to Set A1. Both sets were able to correctly distinguish even the samples from IPF like diseases Sarcoidosis and NSIP from IPF with 100% success.

Moving a step further, the identified genesets were considered for classification by implementing a support vector machine (SVM) model based on the gene expression data. Details of implementations are given in the methods section and associated supplementary methods. The average accuracy over 10 different randomly built models was observed higher for Set A with 92.72% compared to 91.03% accuracy observed for Set A1 (Supplementary Figure S4). Also, the consistency and robustness of the classifiers with Set A genes was superior with higher average Matthews correlation coefficient (MCC) value of 0.85. Though, the performance difference between Set A1 and A is not very high, it is desirable to consider Set A as a better IPF marker set than A1 due to its shorter size and greater conservation across the available experimental data. Detailed results representing Area under curve (AUC), accuracy, specificity, sensitivity and MCC are given in Supplementary Table S1 and Figure S6. Also, it is noteworthy that the Sarcoidosis and NSIP individuals were classified with high accuracy as non-IPF cases (Supplementary Figure S6). This all clearly underlines that the recognized genesets are strong IPF markers, which could additionally be important in the elucidation of underlining molecular systems involved in IPF. Most of these genes were found associated with the process of lung development, maintenance, immune system signaling, collagen metabolism, Extracellular matrix (ECM) deposition, lipid metabolism, and cell-cell interactions, observations well supported by previous studies (Supplementary Table S2). An expression based classification and IPF classification tool has been provided at the associated portal. Considering the dependence on lungs biopsy samples to derive the expression data, the application of such classification system for IPF diagnosis may have practical limitations in most settings. However, it could be useful in high resource settings, where distinction between IPF and other IIPs is critical. In these settings, IPF patients may be referred to a lung transplant program, while NSIP or Sarcoidosis may be treated with high doses of steroids. Open lung biopsies are not uncommon in this setting. Further, such a classification system displays the possibilities for future and could be useful in revalidation processes where the initial clinical diagnosis is discordant with the further clinical course.

After identifying the precise markers for IPF, the next objective was to define the system level map for IPF. Set A might be useful as a prominent molecular marker set for IPF. However, for pairwise comparison across the above mentioned five high throughout studies, a good consensus was observed for common DEGs (Supplementary Figure S1C,D). Presence of common genes across two totally different experiment sets may also be considered credible enough, though with lesser confidence than Set A genes. Considering such genes becomes useful to develop a system level map. With this view a new set of DEGs was created where every gene was found common to at least two different experimental studies. This resulted into a set of 737 DEGs, called Set B, the superset of Set A.

### The regulatory components in IPF: Transcription Factors (TFs) and miRNAs

Previous studies provided some limited details on TFs with respect to IPF^18–20^. A total of 31 DEGs in the above mentioned Set B were found as TFs. Among these, binding sites for 24 TFs were confirmed from literature and repository on experimentally validated TF-target interactions. Functional analysis emphasized on the fact that up-regulated TFs-target genes were associated with ECM-receptor interaction, Integrin family cell surface interactions, IGF-1 and TGF-β signaling pathways. TFAP2A was found regulating highest number of genes in case of upregulated TFs. Similar analysis was done for the down-regulated TFs where CEBPD was found targeting maximum genes (Supplementary Figure S7A,B). In general, processes like fatty acids metabolism and immune system regulation were found targeted by the down-regulated TFs, concurring well with previous findings on downregulated genes (Supplementary Tables S3, S4 and S5).

miRNAs are one of the most important regulatory components of cell system. miRNAs like miR-29b, miR-26a and let-7 were found associated with IPF^21–23^. However, it was quite evident that insufficient full coverage high throughput experiments have been done for miRNAs while studying IPF. This becomes more conspicuous when one looks for more sensitive method like sRNA-seq based studies where a big void exists. In the current study, high throughput data generated from three MA based studies (Cho *et al*., Yang *et al*., Milosevic *et al*.)^9, 11, 24^ were considered for differential expression analysis of miRNAs in IPF, initially. However, no reliable sRNA-seq study was found for IPF. To overcome such scarcity, a strategy was employed where an RNA-seq data^13^ generated for longer RNA like mRNAs was utilized to indirectly derive the expression of miRNAs. Details are given in the methods section.

The relation between the precursor miRNA expression obtained from RNA-seq was compared with the same for mature miRNA expression obtained through MA. It was observed that most of the mature miRNAs displayed strong positive correlation for the precursor derived expression using RNA-seq data. A total of 54 overexpressed and 82 underexpressed miRNAs were detected (Supplementary Figure S8). An overlap was observed for 53 downregulated and 37 upregulated microRNAs from previously reported studies. 42 novel miRNAs (16 up and 26 down) were identified in this study (Supplementary Table S8). 53 out of 54 upregulated and 75 among 82 downregulated miRNAs were found potentially targeting 5,969 and 7,728 genes, respectively. Experimental support was obtained for 818 interactions from all experimental data, including Cross Linking Ligation and Sequencing of Hybrids (CLASH) or Crosslinked Immunoprecipitation (CLIP) analysis. Experimentally supported miRNA interactions had 43 upregulated and 59 downregulated miRNAs, targeting 235 and 428 genes, respectively. Compared to previous studies^9, 11, 24^, the finding on miRNAs is more robust and elaborated due to inclusion of data from multiple experiments including next generation sequencing. miR-539 (overexpressed) and miR-30 family (underexpressed) were obtained as targeting the maximum number of genes (Supplementary Table S9). The interesting question yet to resolve was that how these regulators work together to define the molecular systems of IPF. The following sections address the same.

### Gene regulatory network (GRN) and potential Feed-Forward Loops (FFLs) in IPF

Co-regulation of miRNAs and TFs on same target genes was found to be most common in mammalian genomes^25^. Network motifs in the form of 3 and 4 nodes recurring circuits, referred as FFLs, are considered important in understanding molecular mechanism of disease through capturing the disease expressing network circuits^26–29^. Realizing the importance of the combined effects of various regulatory components, IPF specific network motifs were studied in terms of potential FFLs and concerted regulatory orientations in IPF. While motifs other than FFL are also likely to be important in IPF, here we focused exclusively on FFL because of their instability and potential for dysregulation.

For Set A based potential FFLs, an important observation was that two miRNA-FFLs were consisted of miR-210 targeting matrix metalloproteinase gene (MMP16) in combination with TFs MYOCD and RUNX2. MMP16 is known to be involved in collagen bundle assembly which was observed as upregulated due to downregulation of miR-210 in IPF. This gives explanation for the observation from the previous study which reported that MMP16 is characteristically upregulated in IPF^30^. Another important observation was the identification of miR-30 family downregulation which goes in line with a previous study^23^. miR-30 was found regulating highest number of potential FFLs, mainly consisting of Tetraspanin, involved in alveolar epithelial integrity^31^, PTGFRN, involved in fibroblasts proliferation and collagen production^32^ as well as SFRP2, a modulator of Wnt signaling and a chemokine gene CXCL14, responsible for their overexpression during disease in combination with differentially upregulated TFs BHLHE22 and SIX4.

TF-FFLs for larger network of Set B was composed of 49 miRNA-gene, 63 TF-gene and 32 TF-miRNA interactions (Supplementary Dataset 1). Within TF-FFLs, a differentially downregulated TF, NFE2 (antioxidant enzymes regulator), emerged as an important regulator, targeting crucial genes for IPF phenotypes (FZD5, HHIP, CRTAC1 and EPB41L5) through direct targeting and via upregulation of miR-214. Interesting finding from this study was the observation of potential regulatory circuits having five miRNA clusters. Clusters of [miR-181a, miR-181b], [miR-93, miR-106b], [miR-17, miR-18a, miR-92a] and [miR-30b, miR-30d] were differentially downregulated whereas cluster of miR-133a and miR-1 was upregulated in IPF. Down regulated miRNA clusters were associated with genes involved in Wnt, p53, Jak-STAT, PI3K-Akt, Prolactin signaling and cell adhesion molecules (CAMs) whereas upregulated miRNA cluster was found targeting genes of Fructose and mannose metabolism, cAMP, Hedgehog, PPAR, AMPK signaling, Sphingolipid and fatty acid metabolism. In this way, several network motifs in the form of potential FFLs were identified regulating mainly the process of extracellular matrix organizations, apoptosis, cell proliferation and fatty acid metabolism. Besides having the potential FFL view, it was evident that for many miRNAs multiple targets existed while for several genes multiple targeting miRNAs existed.

Gene regulatory networks were constructed based on the involvement of several common DEGs in IPF, using various network parameters. The networks consisted of 940 nodes connected through 20,207 edges for the set of 737 genes (Set B), and 256 nodes joined via 1,663 edges in the subnetwork of 39 genes (Set A), depicting various important nodes of multiple connectivity. Genes FZD5 along with KCNMA1 involved in repolarization of membrane potential were found as the most important nodes containing highest in-degrees in the GRN of Set A. KCNMA1 gene was observed upregulated in IPF contrary to FZD5. The observations with KCNMA1 is critical because smooth cell/myofibroblast activity is enhanced during IPF where KCNMA1 has a stake. On the other hand, FZD5 is involved in several biological pathways such as Wnt signaling, Hippo signaling and was found downregulated in IPF. Hub gene FZD5 was observed targeted through upregulated miR-155 whose upregulation was supported by a previous study in favor of Epithelial to mesenchymal transition (EMT) process^33^. The underexpressed miR-627 and miR-30 families were observed as critical regulatory hub miRNAs responsible for overproduction of mucin type O-Glycans, increased ECM accumulation and stimulation of mTOR signaling, Cytokine-cytokine receptor interaction, Chemokine signaling and cardiomyopathy. Similarly, upregulated hub miRNA miR-539 was observed targeting the genes involved in steroid biosynthesis (Supplementary Tables S9 and S11). In overall, the upregulated DEGs in the GRN were enriched in ECM receptor interaction, p53 signaling pathway, ABC transporters and CAMs. The downregulated DEGs were found enriched in fatty acid metabolism and AMPK signaling pathways. Detailed discussion on this section is covered in supplementary discussion section.

### Pathway crosstalk analysis reveals compromised and benefited systems in IPF

Crosstalking pathways were identified by integrating gene expression and genome wide Protein Protein Interaction (PPI) data. A total of 56 pathways crosstalks were found for Set B genes (Supplementary Dataset 2), most of which very clearly captured the potential causes and communications responsible for the IPF phenotype while displaying the benefited and compromised pathways chains in IPF.

For example, when PPAR signaling pathway crosstalked with cytokine-cytokine receptor interaction and chemokine signaling pathway, transmembrane receptor activity was compromised influencing lipid transport via increased steroid binding and cholesterol transporter activity. Similarly, when the same pathway joined the complement and coagulation cascades, positive regulation of cellular metabolic process was found decreased and processes related to extracellular matrix and structure organization benefited, a well-known phenotype of IPF. Additionally, same process when crosstalked with leukocyte transendothelial migration pathway, extracellular matrix organization activity induction becomes prominent at the cost of signal transduction activity to enhance fibrotic responses, concurring well with an earlier study^34^.

A prominent and critical marker of IPF is TGF-β, a cytokine stimulator. TGF-β signaling pathway crosstalk with Wnt signaling pathway was observed in agreement to the study demonstrating a novel link between these two pathways with turning down of the expression of Dickkopf-1 which acts antagonistically in Wnt signaling pathway^35^. Additionally, same pathway was found in crosstalk with pathways such as leukocyte transendothelial migration, molding of biological system towards processes involved in extracellular matrix organization. Regulation of endopeptidase activity was compromised to enhance cell surface receptor signaling and cell adhesion as a result of crosstalk between Wnt signaling pathway and TNF signaling pathway. This way, crosstalk analysis revealed several dynamic molecular and system level facets associated with IPF. Detailed information covering all pathways crosstalks and their system influence is provided at the companion IPF database.

### An IPF molecular system model

**Figure 1:**
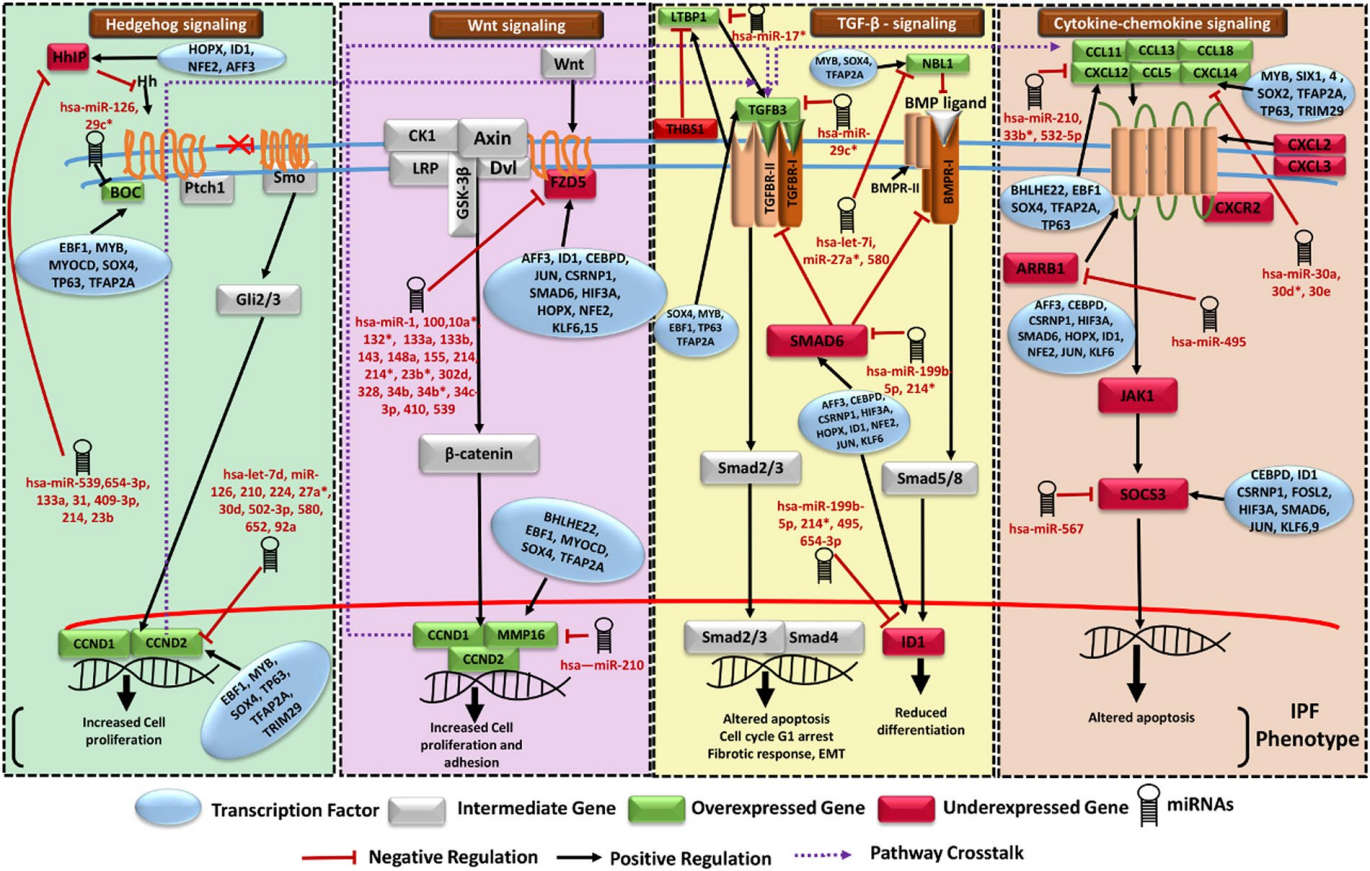
A molecular system model for IPF. It involves four crucial biological pathways (Hedgehog signaling, Wnt signaling, TGFβ signaling and Cytokine-chemokine signaling) having cross-talk with each other. The model describes the mechanism and several regulatory components involved in IPF disease mechanism causing increased cell proliferation, adhesion, reduced differentiation, altered apoptosis and epithelial to mesenchymal transition. DEGs Frizzled 5 receptor, LTBP1, HHIP, BOC, CXCL12, CXCL14, ARRB1, NBL1 and SOCS3 appeared critical in IPF.

A pathway model demonstrating highly influenced pathways in IPF condition was developed (Fig. 1). Increased expression of chemokines (CXCL12, CXCL14) and TGFB3 shown in the model is in agreement with previous studies^36, 37^. TGFB3 binds to its receptor and activates Smad mediated signaling, leading to modulation of apoptosis and G1 stage arrest. TGFB3 and TGFB2 were co-expressing with LTBP1 (Latent TGFβ binding Protein) regulated by THBS1^38^. High expression of TGFB3 induces Smad3 which in association with ATF3 represses inhibitor of differentiation (ID1)^39^ known to be a TGF β responsive gene which negatively regulates cell differentiation. Wnt plays an important role in IPF through binding to Frizzled receptor and further activation of β-catenin. Frizzled (FZD5) appeared downregulated in IPF due to inhibitory regulation by miR-10a*, miR-302d, miR-133b, miR-539, miR-214* and miR-34c-3p (all upregulated) through four potential miR-FFLs with AFF3, HOPX, ID1, KLF6 and one potential TF-FFL (CSRNP1, hsa-miR-133b and FZD5). Genes related to cell proliferation (CCND1 and CCND2) and matrix metallopeptidases (MMPs) involved in cell-cell adhesion appeared upregulated due to combined action of differentially downregulated miRNA (miR-210) and upregulated TFs (TP63, MYOCD) via two potential 3-nodes FFLs (miR-210, TP63, CCND2), (miR-210, MYOCD, MMP16) and a potential 4-nodes FFL (hsa-miR-210, TP63, CCND2, IGF1) for CCND2 and MMPs in IPF condition (Fig. 1). Furthermore, Hedgehog signaling appeared induced through the activation of hedgehog ligand via reduced activity of Hedgehog inhibitory protein (HHIP) due to regulatory network motifs of upregulated miRNAs (miR-409-3p, miR-495 and miR-539) with downregulated TFs AFF3, ID1 and HOPX in disease. BOC gene targeted by miR-126 and miR-29c* (downregulated miRNAs in IPF) positively regulates the Hedgehog signaling through activation of Gli proteins leading to further activation of genes involved in cell proliferation. Additionally, this study revealed pathway crosstalk among four crucial pathways differentially regulated in IPF. Target genes of Hedgehog and Canonical Wnt signaling were found in crosstalk with TGFB3 which further crosstalked with cytokine (CXCL12) stimulating chemokine signaling pathway.

**Figure 2:**
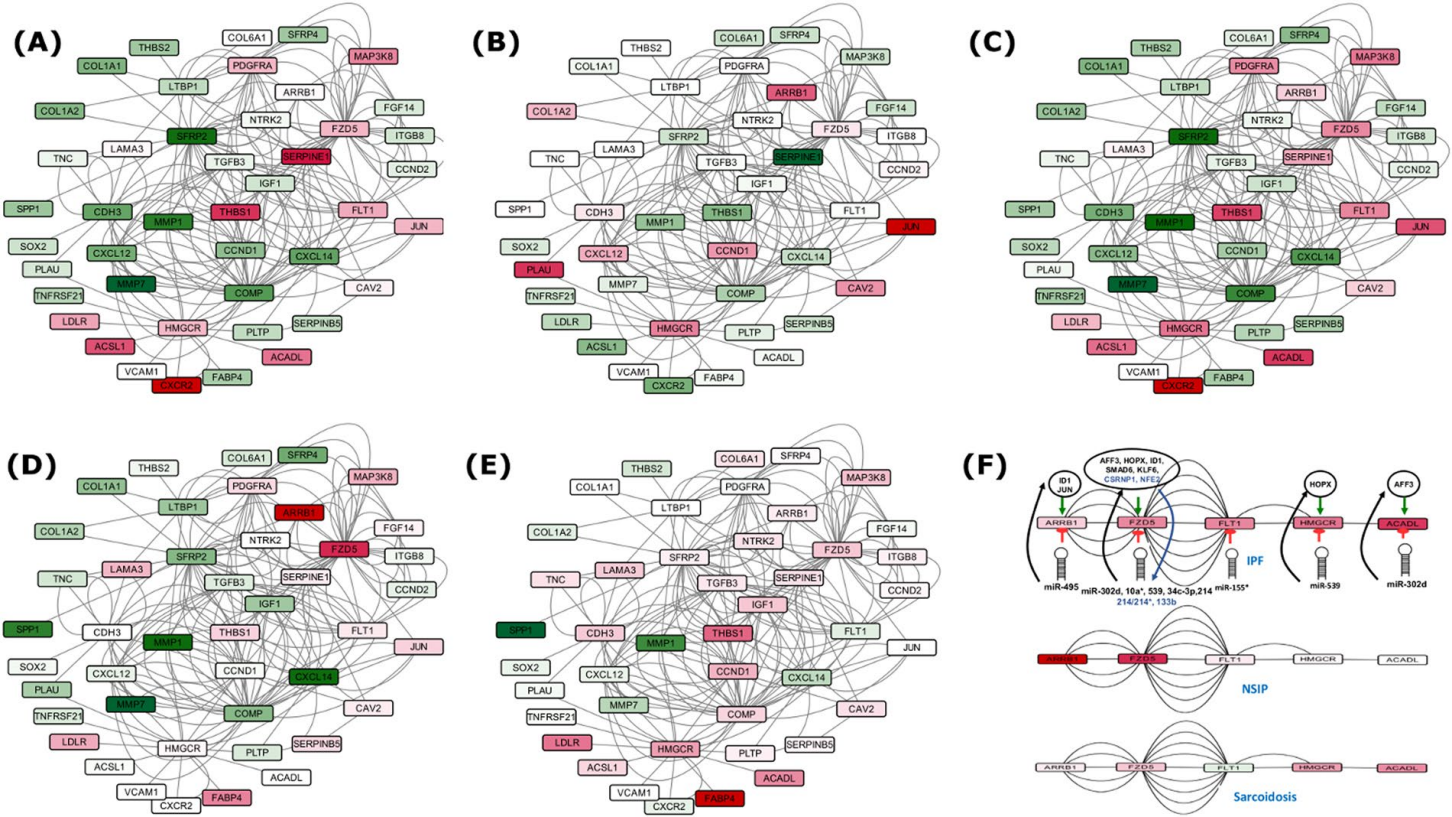
Variation in differential expression of most confident Set A genes along with their first neighbors during IPF progression. (**A**) Normal to Early IPF shifts in differential expression, (**B**) Early to advanced IPF shifts in differential expression, (**C**) Normal to Advanced IPF shift in differential expression, (**D**) differentially expressing genes in NSIP and in (**E**) Sarcoidosis. Green color depicts the degree of overexpression whereas red shows degree of underexpression, (**F**) Critical chain of five serially connected genes (ACADL-HMGCR-FLT1-FZD5-ARRB1) which appeared downregulated in IPF specifically. This chain is broken in Sarcoidosis due to upregulation of FLT1 while in case of NSIP, the chain is not completely formed. Eight TFs and nine miRNAs appeared crucial in regulating IPF through these check points.

To further elaborate the IPF disease progression mechanism, Set A’s DEGs were mapped to the pathways interactome. Differential expression values were derived for associated genes to depict the degree expression changes during IPF progression compared to similar diseases (NSIP and Sarcoidosis). Study revealed a drastic change taking place from normal to early IPF stage, clearly underlining the processes which distinguish IPF from the rest (Fig. 2A–E). The matrix metalloproteinases gene exhibited highest expression shift, and appeared among the most important system chains along with SERPINE1, CXCR2, Tenasein C, FABP4, and CCND1. Among all these major observations for IPF, a system of five serially connected downregulated genes (ACADL-HMGCR-FLT1-FZD5-ARRB1), enriched in regulation of MAPK cascading, potentially targeted by nine different miRNAs and under control of eight different TFs (Fig. 2F) stands out as a crucially IPF specific spot for promising therapeutic interventions for IPF. This all brings a comprehensive, high confidence dynamic system model for IPF. The model proposed here represents a simpler view to underline the important system differences between a normal and IPF condition. This does not take into account the changes in cellular composition of the IPF lung, or the heterogeneity within IPF such that areas of disease are interspersed with normal areas. While models that account for these factors would be superior, it is a difficult task to achieve due to poorly understood factors like remodeling in normal lungs with age, dynamic transitions in cell states and technical challenges in obtaining precisely homogeneous samples.

### The Molecular Systems Database and Information Portal for IPF

The findings and information generated through this study have been converted into a state-of-the-art database and information portal, IPF Information Portal (IPFIP), the resource for this disease, freely available online at http://14.139.59.221/ipf and http://scbb.ihbt.res.in/SCBB_dept/Software.php.

## Conclusion

After analysis of a large volume of high throughput genomic data, a set of 39 differentially expressed genes (DEGs) emerged critical to distinguish IPF. Experimentally validated and high confidence data with multiple evidences were used to reconstruct the system model for IPF, incorporating various PPI and regulatory interactions. Careful analysis of the networks identified several critical potential FFLs as well as compromised and benefited routes in various pathways, marking the phenotype of IPF. A system of serially connected five genes, eight TFs and nine miRNAs appeared exclusive to IPF, which could be promising for therapeutic interventions. In future, the expression of associated miRNAs in this system could be controlled in order to verify their potential role as therapeutic targets for IPF to design therapeutic agents. Finally, this study has generated a state-of-the-art database on IPF where the involved components can be analyzed and visualized in a highly informative manner. This database would be very useful for any molecular systems research on IPF.

## Materials and Methods

### Data processing and analysis

**Figure 3:**
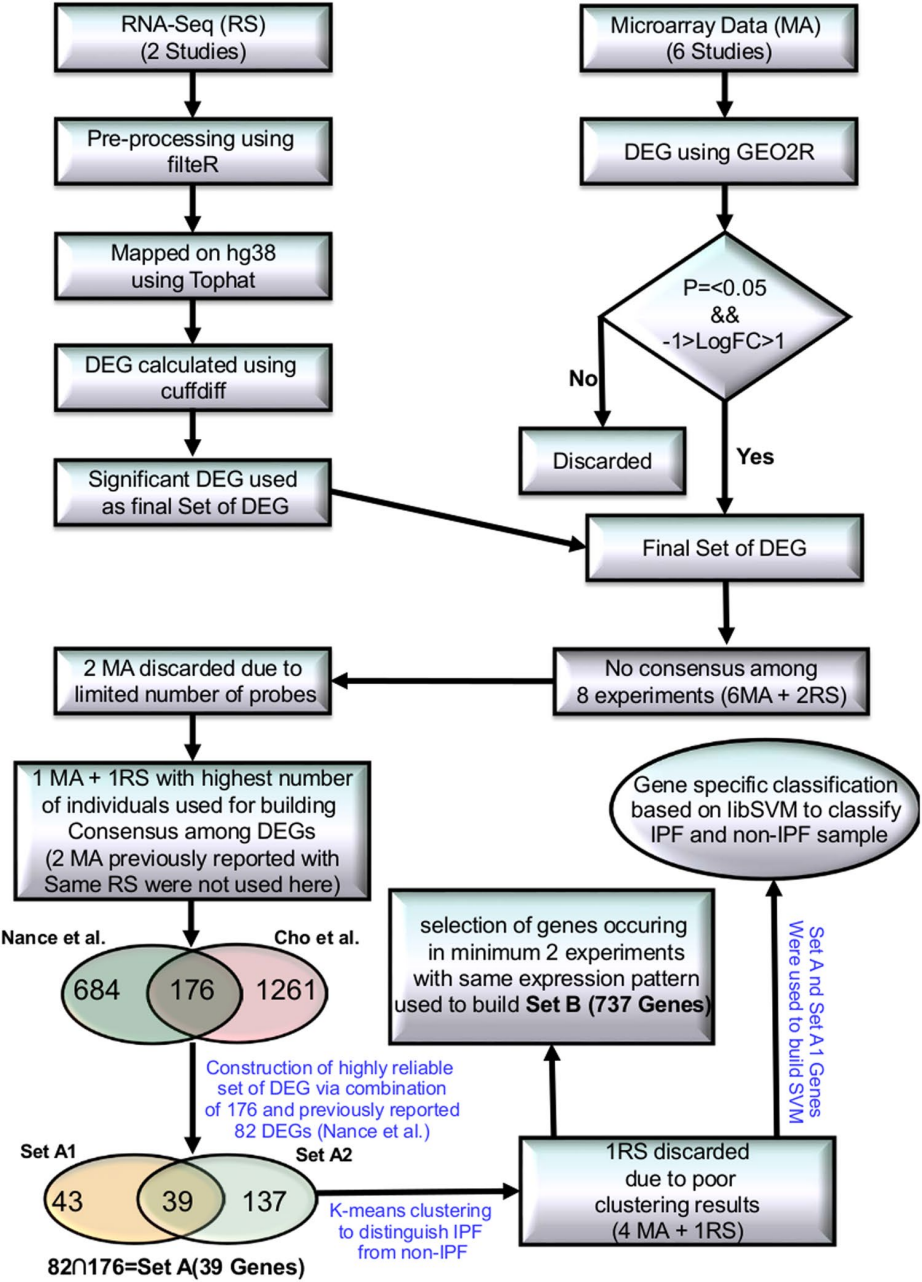
Protocol showing various steps in high-throughput data processing. Two MA studies were discarded due to less number of probes. Initially, Set A2 was constructed using one MA (GSE21411)^9^ and one RNA-seq (SRP033095)^13^ study. Set A was developed from overlap of Set A1 and Set A2. To construct Set B, DEGs were chosen so that they occurred in least two different high throughput studies. A SVM based classification model was generated for using Set A to distinguish IPF sample from non IPF ones.

Fourteen high throughput studies were included containing six IPF MAs (GSE31934, GSE10921, GSE21411, GSE24206, GSE32537, GSE35147)^7–11, 40^, two RNA-seq (SRP010041, SRP033095)^12, 13^, three miRNA MA (GSE21394, GSE32538, GSE27430)^9, 11, 24^, two MAs for Sarcoidosis (GSE16538, GSE19976)^41, 42^ and one MA of NSIP (GSE5774)^43^ (Supplementary Table S12). All initial data from MA and RNA-seq based high throughput studies were collected from Gene Expression Omnibus (GEO) and Sequence Read Archive (SRA). RNA-seq data was cleaned using filteR^44^. TOPHAT 2.1.0^45^ was used for mapping RNA-seq reads across the human genome (hg38) with default parameters. The alignment results were saved in BAM format. CUFFLINK-CUFFDIFF tools were used for expression analysis for RNA-seq data. The work flow of IPF high-throughput data processing to generate high confidence gene sets is given in Fig. 3. For Micro-array gene data, GEO2R (http://www.ncbi.nlm.nih.gov/geo/geo2r/) tool containing R, Biobase^46^, GEOquery^47^ and limma^48^ packages was used with some basic changes in Rscript. Based on the information derived from the clustering of different gene sets with varying number of DEGs (detailed in the result section), set of feature genes was formed. The relative genome wide normalized expression values of these feature genes were used for vector transformational representation of each individual sample considered in this study. Features expression transformation into Z-score for each experimental dataset was done to bring the expression values on same scale irrespective of their platform and experimental design. The classification was implemented with 5-fold cross validation using LibSVM^49^.

### Identification of IPF specific transcription factors and miRNAs

DEGs having TF activity were identified. Sequences of DEGs were downloaded from Ensembl and were searched for homology in UniProt and AnimalTFDB 2.0^50^ using BLASTX with the E-value threshold of 1E-05. Binding sites analysis was done using PROMO (TRANSFAC v8.3)^51^, CHIP-seq (ReMap)^52^ and manual curations. As the TFs activate the gene expression, positive expression correlation is expected between the TF-target gene pair. Z-score normalized expression values were used to find the correlation between TF and target genes. For selecting the significantly enriched categories, p-value cut off (0.05) with Benjamini-Hochberg test adjustment was applied.

miRNA expression analysis was done using four high throughput studies, mentioned above. Interestingly, so far, no small RNA-seq study has been done for miRNAs with respect to IPF. In this study the RNA-seq reads were utilized to detect the active miRNA regions of genome. The overlapped reads with miRNA precursor regions (taken from miRBase V21) were expected to express along with other coding genes. Such mapping reads were capable to provide the expression information of the precursor miRNAs, which in turn indicate the expression level of mature miRNAs. Differentially expressed miRNAs were selected using log_2_fold change (> = 1) considering Reads Per Kilobase of transcript per Million (RPKM) values in both conditions, whereas differentially expressed miRNAs (DEmiRs) from MA studies were selected using GEO2R (p-value < 0.05). miRNAs which were having at-least two or more studies with same differential expression status were taken as final set of DEmiR. miRNAs from RNA-seq approach were preferred over MA in case of contradictory differential expression status (Supplementary Dataset 3). The filtered DEmiRs were analyzed for their target genes using TargetScan^53^ supplemented with experimentally validated interactions data from CLASH and CLIP-seq using miRTarBase version 6.0^54^ while considering a negative expression correlation (“*r*” < = −0.7) for each miRNA-target. A work flow for miRNA analysis is provided in Supplementary Figure S8.

### IPF mediated regulatory network construction and motifs identification

Regulatory relationships in form of miRNA-gene, miRNA-TF, TF-gene and TF-miRNA were considered for generation of regulatory network to unveil disease mechanism in IPF. Differentially expressed miRNAs, genes in IPF condition with respect to normal patient and TFs controlling both were retrieved for deciphering all such relationships. All types of interaction pairs were merged and unified to construct IPF-specific regulatory network. TF and miRNA regulatory network modules having various potential FFLs were derived at *k*-threshold of 3 using clique percolation algorithm, CFinder^55^. Potential FFLs are made of interconnected complete subgraph of TFs, differentially expressed miRNAs and genes. Variations in the type of potential FFL occur due to regulatory interaction between TFs and miRNAs. The work flow for the construction of TF-miRNA mediated regulatory network and statistics of three and four node FFLs for the three sets is illustrated in Supplementary Figure S9A and B, respectively.

IPF specific potential FFLs were evaluated for their significance in comparison to randomized networks. For this purpose, we compared real regulatory networks to randomized ones, preserving same degree as for the real one to estimate the probability that an FFL appears in the randomized networks. 1000 randomized networks were built, implementing edge swapping algorithm present in igraph^56^ R-package. The degree was kept same as for real regulatory network. All potential FFLs in each of the regulatory network were statistically analyzed for their significance while comparing to 1000 randomized regulatory networks. Only those FFLs were considered further for the depiction of regulatory mechanism in IPF which were found statistically significant at 5% level.

Network properties of built TF-miRNA derived regulatory networks were extracted as a consequence of their significance. Cytoscape version 3.1^57^ was used for network visualization and analysis of various network parameters such as degree connectivity, betweenness and closeness centrality etc. Hub miRNAs and TFs were identified by sorting their outdegrees in descending order and top five hub components in each category were separated. Hub IPF genes were determined on the basis of indegree distribution.

### Pathway crosstalk analysis for IPF

Set B DEGs were utilized for pathway crosstalk network reconstruction. Pathways information related to such genes were retrieved from KEGG database. Human protein interaction data was downloaded from STRING v10 repository^58^ of functional protein interaction. A global pathway crosstalk approach was utilized to decipher IPF mediated pathway crosstalk^59^. To determine the background distribution, each pathway was randomized via shuffling of genes with genes having same degree in PPI database. Randomization process was performed 1000 times and protein interaction count was calculated for all randomized pathway pairs. Protein pairs which scored significant p-value against the random model pair occurrence were considered as cross talk pairs.

**Figure 4:**
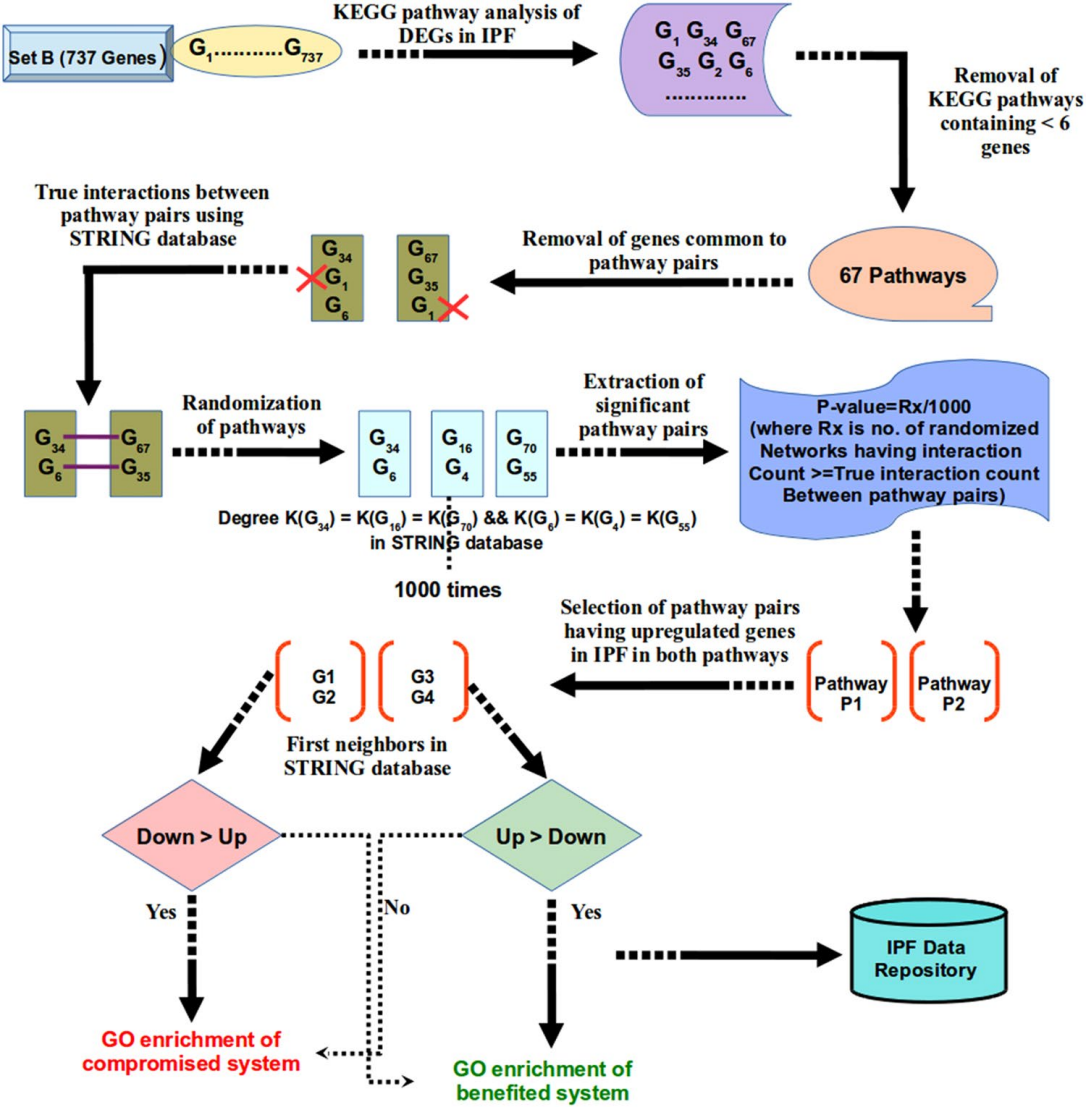
Pipeline depicting global pathway crosstalk network generation protocol for DEGs. Crosstalk network analysis starts by filtering out the pathways containing less than six genes, considering sufficient number of genes to address biological relevance of analysis. Protein interactions occurring between all pathway pairs were counted. Every pathway pair was randomized 1000 times and protein interaction counts in real network were compared with randomized ones. Significant pathway pairs were further analyzed to obtain the benefited and compromised chains in IPF specific pathway crosstalks.

The obtained significant pathway pairs in crosstalk were filtered for those pairs having differentially up regulated genes in both pathways. First neighbors of these DEGs in STRING database containing confidence of 500 (covering most of the experimentally validated protein-protein interactions) were chosen for further analysis. Gene ontology enrichment analysis was done using GO-TermFinder^60^. Gene sets were extracted for benefited and compromised system chains due to pathway crosstalk in the disease condition. Detailed illustration representing pathway crosstalk network analysis pipeline in IPF is shown in Fig. 4. The full method details have been made available in the supplementary methods.

### IPF Information Repository

The portal is implemented using advance libraries of HTML5. Portal homepage and background pages are developed in HTML5/CSS, JQuery/JavaScript with support of Bootstrap packages. Portal work flow is described in Supplementary Figure S10. Background connection is established by PHP version 5.2 with PERL version 5.18 and shell scripting. MySQL version 5.7 database connection is established through the PHP. The database provides the facility for IPF sample identification from expression data, crosstalk and network analysis and can be searched for genes, miRNAs and important pathways. All the interaction can be visualized in selective mode. Common target analysis is also activated for miRNAs which can be extended to the network. All the network elements can be selected for pathways and functional enrichment analysis. The analysis can be done in comparative mode. The network relationships between nodes and edges are structured into JSON file via in-house built PERL script. A set of full network JSON file is loaded into D3, a web based visualization open library. Network properties are represented into html tables using JavaScript for gene regulatory and protein-protein interaction networks. Query sent by a user from HTML pages is processed through PHP in the background and retrieves results of respective sections (like search, network, classification of IPF samples etc.).

## Electronic supplementary material

Supplementary Information

Supplementary Dataset 1

Supplementary Dataset 2

Supplementary Dataset 3
