# Supplementary material for: Detecting the Molecular System Signatures of Idiopathic Pulmonary Fibrosis through Integrated Genomic Analysis
Source: Sci Rep. 2017 May 8;7:1554. doi: 10.1038/s41598-017-01765-6 (PMC5431532; doi:10.1038/s41598-017-01765-6)
Supplement: Supplementary file 1 — Supplementary Information [file 41598_2017_1765_MOESM1_ESM.pdf]

Supplementary Information

Detecting the Molecular System Signatures of Idiopathic Pulmonary Fibrosis through Integrated Genomic Analysis

Indu Gangwar[1Ɨ3], Nitesh Kumar Sharma[1Ɨ3], Ganesh Panzade[13], Supriya Awasthi[1], Anurag Agrawal[2], Ravi Shankar[13]*

Affiliations:

[1] Studio of Computational Biology & Bioinformatics, CSIR-IHBT, Palampur (HP), India.

[2] Lung Diseases Translational Research Group, CSIR-IGIB, Mall Road, Delhi, India.

[3]Academy of Scientific and Innovative Research (AcSIR), CSIR-Institute of Himalayan Bioresource Technology (CSIR-IHBT) Palampur (HP), India

Ɨ Contributed Equally

***Corresponding Author:** *ravish@ihbt.res.in*

Supplementary Methods

High throughput data and processing

Each microarray experiment's data was categorized into two classes, Normal and IPF individuals, while excluding all other specific condition. P-values were adjusted using Benjamini & Hochberg FDR. As in practice, genes with significant P-value (<0.05) and -1<LogFC >1 were considered as differentially expressed genes. Genes which displayed inconsistent and opposite expression status across the samples for any given condition were removed and only those genes were considered which exhibited single consistent trend in any given experiment for the given condition. This removed noise in the micro-array data. Z-score transformation of the expression data was done for all kind of platforms, in order to maintain a single comparable scale.

Classification of IPF genes

For classification purpose, different datasets were created for training and testing using a support vector machine approach. A total of 159 IPF cases worked as positive samples while a total of 112 normal, sarcoidosis and NSIP cases were considered as negative non-IPF cases. For each considered gene set, samples were randomly distributed into training and testing model 10 times, each time yielding different training and testing set pair and model. This was done to ensure that the performance of the classifier was not biased towards only certain combination of samples.

Initially, 271 individuals were considered from five different IPF experiments (169 from Yang et al., 23 from Meltzer et al., 17 in Cho et al., 8 in Sanders et al. and 15 in Nance et al.), 27 from Sarcoidosis (Crouser et al. and Lockstone et al.) and 12 from NSIP (Yang et al.), making a total of 159 IPF and 112 non-IPF individuals. While building the training models, the non-IPF set also included 11 different Sarcoidosis individuals, and 6 NSIP individuals as a part of the non-IPF training dataset along with the control and normal individuals. The remaining 10 Sarcoidosis and 6 NSIP instances would go to the non-IPF test set. This way, 136 individuals having 80 IPF and 56

non-IPF individual were considered for building each model in a mutually exclusive manner in order to avoid any bias. To build random model for each, both the sets (A1 and A), 80 IPF samples were picked randomly as the positive set for the training set. The remaining 79 IPF instances were used as test set. Same was followed for non-IPF instances where 56 individuals were used as negative set for the training and remaining 56 non-IPF instances for testing. This kind of random selection based set pairs were created 10 times to build 10 different models using Radial basis function (RBF) kernel with optimized cost and gamma factors. Each of these models was tested over the corresponding test set.

LibSVM with RBF kernel and optimized cost and gamma factor was run. The models were checked for accuracy, sensitivity and specificity on different randomly generated test sets. Receiver Operator Characteristics plots with Area Under curve were made using ROCR[1] package of R. Matthews correlation coefficient (MCC) was calculated to measure perfection of SVM based classification.

Following equations were used to measure the classifier's performance:

$$Sensitivity = (TP/(TP+FN))*100$$

$$Specificity = (TN/(TN+FP))*100$$

$$Accuracy = (TN+TP)/(TP+TN+FP+FN)$$

$$MCC = (TP*TN)-(FP*FN)/\sqrt{(TP+FP)*(TP+FN)*(TN+FP)*(TN+FN)}$$

where TP= True Positive, TN= True Negative, FP=False Positive and FN= False Negative

Further system wide analysis was done for three datasets (SetA and SetB) as they were found to be most confident differentially expressed sets.

AUCs for above mentioned sets for each of the 10 instance-set pairs were evaluated and scored in the range of 0.92 to 0.99 (Supplementary Figure S6), clearly suggesting robust modeling of IPF by these gene-sets.

TRANSFAC and CHIP-Seq identified TF-target interactions

Besides the manual curation, the approach for the identification of transcription factors involved two more methods: Scanning through 1) TRANSFAC v8.3 and 2) CHIP-seq data. Identification was done for both sets (SetA, B). The first method includes the identification of potentially TF regulated differentially expressed genes, their 2kb upstream promoter regions up to first exon were identified and were searched for transcription factor binding sites using PROMO 3.0. This tool incorporates TRANSFAC database (v8.3) having largest available collection of eukaryotic factor specific weight matrices. TFBS and transcription factor associated with them were identified on the basis of sequence similarity to the matrix. Similarity threshold of 85% was applied to obtain high quality results. The Second method was based on the availability of TF peaks data from regulatory map of transcription factor binding sites (ReMap) repository as it contains validated transcription factor CHIP-seq peak data from well-established repositories ENCODE and GEO. Available transcription factor peak bed files for all 237 transcription factors were downloaded from this repository and their binding was checked across the target genes in both the data sets using bedtools. Details related to the TF-target interactions in the regulation of differentially expressed genes is covered in supplementary Table S6 and list of identified TF is given in supplementary Table S7.

miRNA data

So far three microarray studies have been published for miRNAs (GSE27430, GSE21394 and GSE32538). The study has been done using all these high-throughput studies and a RNA-seq study data. Details are covered in the main text.

Statistical significance of potential Feed-Forward loops

Let suppose two edges E1 and E2 are connecting nodes N0 to N1 and N4 to N5, respectively. These

were chosen randomly and rewired such that nodes were swapped constructing edges E1 =(N0-N5) and E2= (N4-N1). P-value of significance was calculated as proportion of randomized networks having particular FFL count greater than or equal to its number in real network to the total number of randomized networks (1000 in our study).

Network properties of gene regulatory network in IPF

Degree of a vertex/node in the network is the number of edges connecting to it. High degree nodes are characterized as hub nodes in the network. Centrality estimates the importance of node in a network. Betweenness centrality of a node is defined as the total occurrences of node in form of a bridge across shortest path of two other nodes. Farness of a node is a measure of sum of distances of the node with all other nodes and reciprocal of it is defined as the closeness centrality of the node. Tendency of a node in a graph to remain clustered together is referred as clustering coefficient whereas average shortest path length is described as mean value of steps through shortest paths of all potential node pairs of network. Indegree and outdegree terms are defined for directed networks in form of edges converging towards the node and diverging from the node respectively.

Supplementary discussion

IPF specific GRN

IPF associated TF-miRNAs mediated regulatory networks were reconstructed via merging of significant potential FFLs. 15 differentially downregulated genes and 18 differentially upregulated genes were observed regulated by both TFs and miRNAs, forming network motifs in Set A. 22 upregulated miRNAs and 19 downregulated miRNAs in IPF were found regulating IPF DEGs via association with 42 TFs incorporating 16 DETFs in IPF (CSRNP1, NFE2, AFF3, BHLHE22, HOPX, ID1, JUN, KLF6, MYB, MYOCD, SIX1, SIX4, SMAD6, SOX2, TFAP2A and TP63) (Supplementary Figure S11). Upregulated DEGs forming significant potential FFLs in these sets

were enriched in ECM receptor interaction, p53 signaling pathway, ABC transporters and cell adhesion molecules (CAMs). The downregulated DEGs were found enriched in fatty acid metabolism and AMPK signaling pathways. Enhanced extracellular matrix deposition and cell adhesion was observed as a characteristic feature of IPF lungs. Previous studies had reported p53 upregulation causing G1 arrest of cell cycle combining chronic DNA damage in IPF disease condition[33]. Fatty acid metabolism affects function of alveolar epithelial cells, which are responsible for maintaining lung homeostasis. Its deficiency stimulates lung injury in bleomycin induced mice resulted in induction of apoptosis and TGF-β expression[34]. All these previous observations concur with the findings here.

Table S1: Accuracy, sensitivity, specificity and MCC values for sets A1 and A in contrast to ten different random models

Set A								
	**TP**	**FN**	**FP**	**TN**	**Accuracy**	**Sensitivity**	**Specificity**	**MCC**
**Shuffle 1**	78	2	6	50	94.12	97.5	89.29	0.88
**Shuffle 2**	78	2	5	51	94.85	97.5	91.07	0.89
**Shuffle 3**	76	4	8	48	91.18	95	85.71	0.82
**Shuffle 4**	77	3	4	52	94.85	96.25	92.86	0.89
**Shuffle 5**	78	2	8	48	92.65	97.5	85.71	0.85
**Shuffle 6**	78	2	7	49	93.38	97.5	87.5	0.86
**Shuffle 7**	75	5	10	46	88.97	93.75	82.14	0.77
**Shuffle 8**	80	0	7	49	94.85	100	87.5	0.9
**Shuffle 9**	77	3	11	45	89.71	96.25	80.36	0.79
**Shuffle 10**	79	1	9	47	92.65	98.75	83.93	0.85
**Set A1**								
	**TP**	**FN**	**FP**	**TN**	**Accuracy**	**Sensitivity**	**Specificity**	**MCC**
**Shuffle 1**	76	4	11	45	88.97	95	80.36	0.77
**Shuffle 2**	72	8	9	47	87.5	90	83.93	0.74
**Shuffle 3**	73	7	7	49	89.71	91.25	87.5	0.79
**Shuffle 4**	77	3	8	48	91.91	96.25	85.71	0.83
**Shuffle 5**	79	1	9	47	92.65	98.75	83.93	0.85
**Shuffle 6**	76	4	12	44	88.24	95	78.57	0.76
**Shuffle 7**	78	2	5	51	94.85	97.5	91.07	0.89
**Shuffle 8**	76	4	7	49	91.91	95	87.5	0.83
**Shuffle 9**	80	0	11	45	91.91	100	80.36	0.84
**Shuffle 10**	76	4	6	50	92.65	95	89.29	0.85

	Accuracy Result in GSE35147 (Only involved in Test Set)									
	**Shuffle 1**	**Shuffle 2**	**Shuffle 3**	**Shuffle 4**	**Shuffle 5**	**Shuffle 6**	**Shuffle 7**	**Shuffle 8**	**Shuffle 9**	**Shuffle 10**
**Set A**	100	100	87.5	100	100	100	87.5	100	75	100
**Set A1**	100	75	100	87.5	100	100	87.5	87.5	100	75

Table S2: Functional role and status of Set A DEGs in IPF

Gene Name	Status in IPF	Role in IPF
Caveolin-2 (CAV1/2)	Down	It is a membrane protein which is present in lung fibroblasts and suppress TGFβ induced ECM production but in case of IPF, its decrease causes excessive TGFβ induced ECM production.
CBS	Down	It regulates homocysteine in plasma membrane and deficiency of CBS genes in mouse model already published as one of cause of fibrosis and inflammation
CDH3	Up	It works as a molecular target in various cancer like pancreatic, colorectal and gastric cancer[2]. Upregulation of this gene supports the proliferation and adhesion in IPF lungs
SFRP2	Up	Secreted frizzled related protein 2 works as inhibitor of Wnt signaling pathway and upregulation of this gene inhibits BMP2 gene which is a key factor in collagen maturation and biosynthesis during injury condition[3]
COL14A1	Up	It is a type of collagen, a member of the FACIT (fibril-associated collagen with interrupted triple helices) collagen family. This protein interacts with fibril surface and is directly involved in the regulation of fibrillogenesis.
COMP	Up	Cartilage oligomeric matrix protein (COMP) is a non-collagenous ECM protein, knockdown of this gene inhibits cell proliferation which shows direct impact of this gene under IPF condition[4]
CXCL14	Up	Production of CXCL14 is done by macrophages, fibrotic loci and type 2 alveolar epithelial cells, it's a mucosal chemokine which recruits many immune cell on mucosa to prevent a cell against foreign particle working as an anti-microbial gene[5]
DCLK1	Up	Doublecortin-like kinase 1 (DCLK1) is a member of kinase superfamily and worked as marker for cancer stem cell and directly involved in epithelial-mesenchymal transition to enhance it which is also a key phenotype of IPF[6]
DIO2	Up	Deiodinase iodothyronine type II (dio2) is a member of iodothyronine family which activates thyroid hormone by converting the prohormone thyroxin. From the study, it was found that upregulation of this gene changes in signaling of thyroid hormone which may play a role in development and progression of IPF but direct involvement was not observed in IPF[7]
EPB41L5	Down	Down regulated Erythrocyte membrane protein band 4.1 like 5 (EPB41L5) is used as a biomarker for idiopathic pulmonary fibrosis[8]
FAM167A	Down	Downregulation of FAM167A is a novel finding discovery in this study and was found regulated by miR-27a and transcription factors (ATF3 and VDR). Previous

		studies reported that FAM167A is involved in sclerosis, a specific type of fibrosis, where fibrous deposition in an area occurs which has some sort of flexibility or movement like skin
FZD5	Down	Frizzled class receptor 5 (FZD5) encodes 7-transmembrane domain protein that are receptors for Wnt signaling believed to be the receptor for Wnt5a ligand and downregulation of this gene reduces the angiogenesis[9]
HMGCR	Down	It is a rate limiting factor for fatty acid biosynthesis, and downregulation of this enzyme leads to downregulation of fatty acid biosynthesis.
IGFBP4	Up	Elevated expression of Insulin-like growth-factor-binding proteins (IGFBP4) shows inhibitory effect on canonical Wnt signaling which plays a crucial role in IPF
KCNMA1	Up	Potassium calcium-activated channel subfamily M alpha 1 (KCNMA1) was found to act as muscle relaxant. In IPF condition its expression was elevated and altering lungs muscle cell tone.
KRT17	Up	Upregulated Keratin 17 involved in tissue repair and formation and maintenance of various skin appendages by regulating protein synthesis and epithelial cell growth through binding to the adapter protein SFN.
LTBP1	Up	Latent TGF β protein-1 (LTBP1) was found up-regulated in our study. A previously reported study suggested that alteration of TGF-β pathways alters the extracellular matrix composition in fibrotic lung[10]
MMP16	Up	Matrix metallopeptidase 16 (MMP16) was found upregulated and has an important involvement in the breakdown of ECM in normal physiological processes, like tissue remodeling, reproduction and embryonic development as well as in chronic lung diseases.
PAMR1	Up	Peptidase domain containing associated with muscle regeneration 1 (PAMR1) function is not very well defined but it was upregulated and it is believed that it may play an important role in formation of skeletal muscle.
PCDH7	Up	Protocadherin (PCDH7) belongs to cadherin super family and have role in cell-cell recognition and adhesion. Increased expression of this gene supports elevated cell adhesion process in IPF.
Thy-1	Up	Thy-1 was found upregulated and it is a glycoprotein encoded by cell surface antigens and elevates cell adhesion and cell communication.

Table S3: Differentially expressed TF-target interaction analysis for Set A and Set B genes.

TF- target interaction analysis for a Set B genes					
Differentially expressed genes	Correlation >=0.7	TF	Target gene	Pathways identified from target enrichment p-value	GO-terms P-value
up-regulated (468)	2037 interactions	11	381	ECM-receptor interaction-(p=1.66E-11) integrin family cell surface interactions-(p=7.86E-16) Focal adhesion-(p=5.01E-10) IGF1 pathway-(p=8.02E-12)	Cell adhesion-(p=2.00E-12) ECM organization-(p=1.50E-12) Growth factor binding-(p=6.40E-05)
down-regulated (269)	1721 interactions	13	241	Metabolic pathway-(p=2.33E-06) Endocytosis- (p=7.42E-06)	Immune system process- p(1.17E-05) response to wounding (p= 1.08E-06) RAGE receptor binding-(p=0.0118)
**TF- target interaction analysis for a Set A genes**					
Differentially expressed genes	Correlation >=0.7	Transcr iption Factor	Target genes	Pathways identified from target enrichment -P value	GO terms P-value
up-regulated (22)	132 interactions	11	21	TGF- β signaling pathway (p=0.0008)	Cell adhesion(p=0.0 317)
down-regulated (17)	110 interactions	13	15	Metabolic pathways (p=0.0005) Metabolism of lipids and lipoproteins (p= 0.0001)	Sterol biosynthetic process(p=7.68 e-06)

Table S4: Differentially expressed up-regulated TF classification and role in IPF

Transcription factor	Class	Family	Expression in study	Function	Target Involvement in up-regulated pathways associated with IPF identified in study	Target genes GO enriched terms associated with IPF identified in study	role in IPF
SOX2- Sex Determining Region Y -Box 2	High mobility group domain (HMG) domain factors	SOX-related factors	up-regulated	proliferation and differentiation	beta1 integrin cell surface interactions, SIP1 pathway, ECM receptor interaction pathway	Cell adhesion, ECM organization.	Imbalanced activity of airway epithelial cells, results in formation of alveolar lesions in IPF[11]
SOX4- Sex Determining Region Y -Box 4	High mobility group domain (HMG) domain factors	SOX-related factors	up-regulated	mesenchymal cell expression	ECM-receptor interaction, Focal adhesion pathway, beta1 integrin cell surface interactions, SIP1 pathway	Cell adhesion, ECM organization, growth factor binding	Higher expression is responsible for mesenchymal formation from EMT[12]
EBF1- Early-B-cell related factor	Rel Homology Region factors	Early B-Cell Factor-related factors	up-regulated	differentiation and maturation of B-cells	ECM-receptor interaction, Focal adhesion pathway	Cell adhesion, ECM organization, growth factor binding	B-cell aggregation[13]
SIX1- Sine oculis homeobox-1	Homeo domain factors	HD-SINE factors	up-regulated	mesenchymal cell formation	beta1 integrin cell surface interactions, IGF1 pathway	Cell adhesion, ECM organization	Higher expression is responsible for EMT transitions[14]
SIX4- Sine oculis homeobox-4	Homeo domain factors	HD-SINE factors	up-regulated	cell proliferation	beta1 integrin cell surface interactions, signaling events mediated by focal adhesion kinase	Cell adhesion, ECM organization	Higher expression enhances the myofibroblast differentiation[15]
TFAP2A-Transcription factor AP-2-alpha	Basic helix-span-helix factors	AP-2 factors	up-regulated	cell growth and differentiation	ECM-receptor interaction, Focal adhesion, beta1 integrin cell surface interactions	Cell adhesion, ECM organization, growth factor binding	upregulation of TFAP2A promotes cell differentiation and important role in TGF-beta induced signaling[16]
MYOCD-Myocardin	Basic-helix-loop-helix factors	ASC-related factors	up-regulated	Cell proliferation and differentiati	ECM receptor interaction, Focal adhesion, beta1 integrin cell	Cell adhesion, ECM organization,	Progressive and irreversible scarring of lung tissue[17]

	(Bhlh)			on	surface interactions	growth factor binding	
TP63-Tumor suppressor protein 63	P53 domain factors	P53 related factors	up-regulated	cell proliferation	ECM receptor interaction, Focal adhesion, beta1 integrin cell surface interactions	Cell adhesion, ECM organization, growth factor binding	Disordered proliferation of epithelial cells results in squamous metaplasia as one of the classified form of honeycomb structure in IPF[18]
TRIM29-Tripartite motif-containing protein29	C2H2 zinc finger factors	Trim-family	up-regulated	cell proliferation and transformati on	ECM receptor interaction, Focal adhesion, P53 signaling pathway	ECM organization, ECM assembly, growth factor binding	Higher expression results in inhibition of apoptosis[19]
MYB-Transcription l activator Myb	Tryptopha n cluster factors	Myb/SANT domain factors	up-regulated	cell proliferation	ECM receptor interaction, Focal adhesion, beta1 integrin cell surface interactions	Cell adhesion, ECM organization, growth factor binding	Enhance myofibroblast formation and differentiation from ECM transition[20]
BHLHE22-Class E basic helix-loop-helix protein 22	Basic-helix-loop-helix factors (Bhlh)	Tal- related factors	up-regulated	cell proliferation	ECM receptor interaction, Focal adhesion, beta1 integrin cell surface interactions	Cell adhesion, ECM organization, growth factor binding	Higher expression is responsible for enhance cell proliferation

Table S5: Differentially expressed down-regulated TF classification and role in IPF

Transcription factor	Description	Class	Family	Expression in study	Function	Involvement in down-regulated pathways associated with IPF identified in study	GO enriched terms associated with IPF identified in study	role in IPF
CEBPD	CCAAT/enhancer binding protein delta	Basic leucine zipper factors (bZIP)	C/EBP family	down-regulated	tumor suppressor function	Metabolic pathways, Endocytosis	Response to wounding, RAGE receptor binding	enhance fibroblast proliferation[21]
CSRNP1	Cysteine rich nuclear protein	AXUD/ CSRNP domain factors	CSRNP factors	down-regulated	tumor suppressor function	Metabolic pathways, Endocytosis	Response to stress, response to wounding	Down-regulation inhibits tumor suppression activity[22,23]
FOSL2	FOSL2	Basic leucine zipper factors (bZIP)	Fos-related factors	down-regulated	cellular proliferation	Metabolic pathways, Endocytosis	Response to stress, response to wounding	Down-regulation inhibits its anti-proliferation activity[24]
HIF3A	Hypoxia-inducible factor-3-alpha	Basic-helix-loop-helix factors (Bhlh)	PAS domain factors	down-regulated	mediates oxidative stress	Metabolic pathways, Endocytosis	Response to stress, response to wounding	Down-regulation leads to hypoxia induce damage[25]
HOPX	Homeodomain- only protein	Homeo domain factors	Paired-related HD factors	down-regulated	developing pulmonary airway	Endocytosis, Metabolic pathways	Response to chemical stimulus, RAGE receptor binding	Down-regulation lead to alveolar damage[26]
ID1	Inhibitors of Differentiation	Basic-helix-loop-helix factors (Bhlh)	ID family	down-regulated	inhibits differentiation	Endocytosis, Metabolic pathways	Response to wounding, RAGE receptor binding	Down-regulation induces impairment and fibrosis[27]
JUN	Jun	Basic leucine zipper factors (bZIP)	Jun-related factors	down-regulated	cell cycle progression and apoptosis	Endocytosis, Metabolic pathways	Response to stimulus, regulation of catalytic activity	Down-regulation induces reduction in apoptosis[24]
KLF6	Kruppel -	C2H2	Three-zinc	down-	differentiati	Endocytosis,	Response	dysregulation of

	like factor 6	zinc finger factors	finger Kruppel-related factors	regulated	on and apoptosis	Metabolic pathway	to stimulus, oxidoreductase activity	apoptosis in vasculature in IPF[28]
KLF9	Kruppel- like factor 9	C2H2 zinc finger factors	Three-zinc finger Kruppel-related factors	down-regulated	Regulates cell proliferation, B-cell proliferation, apoptosis	Endocytosis, Metabolic pathways	Response to stress, response to wounding	Down-regulation inhibits its apoptotic activity.
KLF15	Kruppel- like factor 15	C2H2 zinc finger factors	Three-zinc finger Kruppel-related factors	down-regulated	Cellular proliferation inhibition	Endocytosis, Metabolic pathway	Cellular response to stimulus, carbohydrate binding	Down-regulation inhibits its role as novel inhibitor of VSMCs proliferation[29]
NFE2	Nuclear factor erythroid 2	Basic leucine zipper factors (bZIP)	Jun-related factors	down-regulated	Regulation of megakaryocytic differentiation and regulation of platelet production	Endocytosis, Metabolic pathways	Immune system process, response to wounding, RAGE receptor binding	Down-regulation results in dysregulation of platelet production[30]
SMAD6/ MADH6	Mother against decapentaplegic homolg 6	SMAD/ NF-1 DNA-binding domain factors	SMAD factors	down-regulated	repressor activity	Endocytosis, Metabolic pathways	Response to stimulus, oxidoreductase activity	Down-regulation inhibits its antifibrotic activity[31]
AFF3	AFF3/LAF4	NA	AF4/FMR 2 family member 3	down-regulated	Lymphoid development and oncogenesis	Endocytosis, Metabolic pathways	Response to external stimulus, oxidoreductase activity	Downregulation results in decreased lymphocyte signaling[32]

Table S6: Transcription factor-target analysis using Promo (transfac v8.3) and ReMap

TF-target interaction analysis using PROMO for a Set A genes			
**Differentially expressed genes**	**TF-target interactions Correlation >=0.7**	**Transcription Factor**	**Target Genes**
up-regulated (22)	294 interactions	30	20
down-regulated (17)	152 interactions	24	15
**TF-target interaction analysis using PROMO for a Set B genes**			
up-regulated (468)	4103 interactions	34	375
down-regulated (269)	2976 interactions	34	230
**TF-target interaction analysis using ReMap for a Set A genes**			
**Differentially expressed genes**	**TF-target interactions Correlation >=0.7**	**Transcription Factor**	**Target Genes**
up-regulated (22)	325 interactions	78	21
down-regulated (17)	297 interactions	68	14
**TF-target interaction analysis using ReMap for a Set B genes**			
up-regulated (468)	4748 interactions	97	343
down-regulated (269)	4775 interactions	95	222

Table S7: List of TF regulating differentially expressed genes (A total 107 TFs were identified from both methods (TRANSFAC and CHIP-Seq). Enrichment analysis of their target genes showed the involvement of similar pathways as well as the involvement of same biological and molecular functions as identified in case of differentially expressed transcription factors.)

AR	EZH2	LEF1	RFX5	VDR
ATF1	FLI1	MAFF	RUNX1	WT1
ATF3	FOS	MAFK	RUNX1T1	XBP1
ATRX	FOSL1	MEF2C	RUNX2	ZEB1
BACH1	FOXA1	MEIS1	SMAD1	ZKSCAN1
BCL11A	FOXA2	MTA3	SMC1A	ZNF143
BCL3	FOXM1	MYC	SMC3	ZNF217
BCL6	FOXP2	NANOG	SMC4	
BHLHE40	GABPA	NCOR2	SNAPC1	
BRCA1	GATA2	NFATC1	SNAPC5	
CDK8	GATA3	NFATC2	SPI1	
CEBPA	GATA6	NFKB1	SRF	
CEBPB	GREB1	NKX2-1	STAT1	
CHD1	HDAC2	NOTCH1	STAT2	
CHD2	HMGN3	NR2F1	STAT4	
EGR1	HNF1B	PAX5	SUZ12	
EGR3	HNF4G	PML	TAF1	
ELF5	HOXD10	POU5F1	TAL1	
EOMES	IKZF1	PPARG	TCF4/TCF7L2	
ERG	IRF1	PPARGC1A	NR2F2	
ESR1	IRF4	PRDM1	TEAD4	
ESR2	JUNB	RAD21	TFAP2C	
ETS1	JUND	RARB	THAP1	
ETS2	KDM5B	RBPJ	TP53	
ETV1	KLF4	RCOR1	USF1	

Table S8: Novel, overlapping and contradictory set of DEmiRNAs during IPF

**Novel miRNAs**	
**Upregulated**	**Downregulated**
hsa-let-7c, hsa-miR-23b, hsa-miR-34a, hsa-miR-34c-3p, hsa-miR-34c-5p, hsa-miR-100, hsa-miR-133a, hsa-miR-135b, hsa-miR-302d, hsa-miR-328, hsa-miR-363, hsa-miR-429, hsa-miR-567, hsa-miR-634, hsa-miR-921, hsa-miR-1249	hsa-let-7d, hsa-let-7i, hsa-miR-28-3p, hsa-miR-33b, hsa-miR-139-3p, hsa-miR-146a, hsa-miR-342-3p, hsa-miR-346, hsa-miR-486-5p, hsa-miR-550, hsa-miR-580, hsa-miR-593, hsa-miR-601, hsa-miR-623, hsa-miR-627, hsa-miR-632, hsa-miR-636, hsa-miR-638, hsa-miR-641, hsa-miR-671-5p, hsa-miR-874, hsa-miR-877, hsa-miR-933, hsa-miR-939, hsa-miR-1228, hsa-miR-1287
**Previously known miRNAs**	
**Upregulated**	**Downregulated**
hsa-miR-1, hsa-miR-10a*, hsa-miR-21, hsa-miR-31, hsa-miR-31*, hsa-miR-34b, hsa-miR-99a, hsa-miR-125b, hsa-miR-132, hsa-miR-133b, hsa-miR-143, hsa-miR-148a, hsa-miR-154, hsa-miR-155, hsa-miR-199a-5p, hsa-miR-199b-5p, hsa-miR-205, hsa-miR-214, hsa-miR-214*, hsa-miR-299-5p, hsa-miR-376a, hsa-miR-376b, hsa-miR-376c, hsa-miR-377, hsa-miR-379, hsa-miR-381, hsa-miR-382, hsa-miR-409-3p, hsa-miR-410, hsa-miR-411, hsa-miR-487b, hsa-miR-493*, hsa-miR-495, hsa-miR-509-5p, hsa-miR-539, hsa-miR-650, hsa-miR-654-3p	hsa-let-7b, hsa-let-7g, hsa-miR-17, hsa-miR-18a, hsa-miR-23a, hsa-miR-27a, hsa-miR-29c, hsa-miR-30a, hsa-miR-30b//hsa-miR-30b*, hsa-miR-30c-1*, hsa-miR-30c-2*, hsa-miR-30d, hsa-miR-30e, hsa-miR-33a, hsa-miR-92a, hsa-miR-93, hsa-miR-106b, hsa-miR-125a-3p, hsa-miR-126, hsa-miR-141, hsa-miR-151-3p, hsa-miR-181a, hsa-miR-181b, hsa-miR-181d, hsa-miR-184, hsa-miR-191, hsa-miR-203, hsa-miR-193-3p, hsa-miR-210, hsa-miR-221, hsa-miR-221*, hsa-miR-222, hsa-miR-223, hsa-miR-224, hsa-miR-320a, hsa-miR-326, hsa-miR-342-5p, hsa-miR-345, hsa-miR-362-5p, hsa-miR-375, hsa-miR-378, hsa-miR-422a, hsa-miR-423-5p, hsa-miR-425, hsa-miR-489, hsa-miR-500, hsa-miR-502-3p, hsa-miR-532-3p, hsa-miR-532-5p, hsa-miR-652, hsa-miR-663, hsa-miR-668, hsa-miR-744
**Contradicted miRNAs with previous studies**	
**Our study upregulated**	**Previously known as downregulated**
hsa-miR-29a	hsa-miR-29a
**Our study downregulated**	**Previously known as upregulated**
hsa-miR-92b/hsa-miR-92b*, hsa-miR-324-3p, hsa-miR-765	hsa-miR-92b/hsa-miR-92b*, hsa-miR-324-3p, hsa-miR-765

Table S9: Top ten differentially expressed miRNAs on the basis of target gene count and their functional enrichment.

Upregulates miRNA			
**miRNA**	**Enriched KEGG pathway**	**Target genes**	**P-value of enrichment**
hsa-miR-10a*	Steroid biosynthesis	16	0.000022
	Metabolic pathways		0.0078
hsa-miR-133a	Basal cell carcinoma	19	0.0003
	Endocytosis		0.0035
	Pathways in cancer		0.0089
hsa-miR-23b*	Terpenoid backbone biosynthesis	21	4.51E-008
	Metabolic pathways		0.0000124
	Steroid biosynthesis		0.0000384
	Melanogenesis		0.0011
hsa-miR-302d	Melanogenesis	17	0.0007
	Neuroactive ligand-receptor interaction		0.0051
hsa-miR-31	Terpenoid backbone biosynthesis	16	0.0000135
	Steroid biosynthesis		0.000022
	Metabolic pathways		0.000042
hsa-miR-34b*	Steroid biosynthesis	17	4.91E-008
	Metabolic pathways		0.00000308
	Biosynthesis of unsaturated fatty acids		0.0000306
	Glycine, serine and threonine metabolism		0.000072

	Basal cell carcinoma		0.0002
	Pathways in cancer		0.0072
hsa-miR-34c-3p	Terpenoid backbone biosynthesis	32	0.000000168
	Steroid biosynthesis		0.0000905
	Biosynthesis of unsaturated fatty acids		0.0001
	Metabolic pathways		0.0002
	Basal cell carcinoma		0.0008
hsa-miR-495	Neuroactive ligand-receptor interaction	30	0.0000373
	Pathways in cancer		0.0000752
	Focal adhesion		0.0004
	Endocytosis		0.0086
hsa-miR-539	Terpenoid backbone biosynthesis	36	0.000000241
	Steroid biosynthesis		0.000000513
	Metabolic pathways		0.00000342
	Biosynthesis of unsaturated fatty acids		0.0001
	Basal cell carcinoma		0.001
	Drug metabolism - cytochrome P450		0.0017
hsa-miR-654-3p	Neuroactive ligand-receptor interaction	18	0.0057
	Metabolic pathways		0.0109
**Downregulated miRNA**			
hsa-miR-17*	Protein digestion and absorption	18	0.0005

	ECM-receptor interaction		0.0006
	Amoebiasis		0.0009
	Focal adhesion		0.0031
hsa-miR-210	Hypertrophic cardiomyopathy (HCM)	31	2.97e-05
	Focal adhesion		0.0004
	mTOR signaling pathway		0.0006
	p53 signaling pathway		0.0011
	Protein digestion and absorption		0.0016
	Dilated cardiomyopathy		0.0019
	Salivary secretion		0.0019
	Leukocyte transendothelial migration		0.0032
	Cell cycle		0.0036
hsa-miR-30a	Hypertrophic cardiomyopathy (HCM)	53	0.0044
hsa-miR-30d*	Hypertrophic cardiomyopathy (HCM)	26	1.73e-05
	mTOR signaling pathway		0.0005
	Arrhythmogenic right ventricular cardiomyopathy (ARVC)		0.0009
	Dilated cardiomyopathy		0.0014
	Cell adhesion molecules (CAMs)		0.0029
	Focal adhesion		0.0065
hsa-miR-30e	Cytokine-cytokine receptor interaction	37	0.0014
	Arrhythmogenic right ventricular cardiomyopathy (ARVC)		0.0018
	Hypertrophic cardiomyopathy (HCM)		0.0022
hsa-miR-33b*	Focal adhesion	21	0.0001
	Protein digestion and absorption		0.0006
	ECM-receptor interaction		0.0007

	Cell adhesion molecules (CAMs)		0.0017
	Cytokine-cytokine receptor interaction		0.0066
hsa-miR-345	Hypertrophic cardiomyopathy (HCM)	27	0.0012
	Cell adhesion molecules (CAMs)		0.0029
hsa-miR-532-5p	Leukocyte transendothelial migration	25	0.0019
	Chemokine signaling pathway		0.0049
	Cytokine-cytokine receptor interaction		0.0095
hsa-miR-627	Mucin type O-Glycan biosynthesis	59	0.0007

Table S10: Description of various potential Feed-Forward loops and interaction in IPF-mediated regulatory networks

	Motif	Number of potential FFLs	Genes	miRNAs	TFs	MiRNA-gene	MiRNA-TF	TF-gene	TF-miRNA	Gene-Gene
**Set A** genes network	3node TF-FFL	17	5	7	8	13	-	15	11	-
	3node MiRNA-FFL	246	33	39	35	137	86	161	-	-
	Total	263	33	43	42	143	86	176	11	-
**Set A** genes network	4node TF-FFL	-	-	-	-	-	-	-	-	-
	4node MiRNA-FFL	13	14	5	5	16	5	16	-	5
	Total	13	14	5	5	16	5	16	-	5
**Set B** genes network	3node TF-FFL	65	36	19	21	49	-	63	32	-
	3node MiRNA-FFL	1250	242	64	64	584	153	963	-	-
	Total	1315	243	73	77	607	153	1026	32	-
**Set B** genes network	4node TF-FFL	1	2	1	1	2	-	2	1	1
	4node MiRNA-FFL	93	43	20	19	77	32	112	-	42
	Total	94	43	21	20	79	32	114	1	42

Table S11: Hub components of regulatory network in terms of IPF genes, IPF miRNAs and TFs

IPF Genes	Indegree/ Outdegree	Function	IPF miRNA	Indegree/ Outdegree	TFs	Indegree/ Outdegree	Function
**Set B Genes regulatory network**							
PDE4D	105/1	Alveolar Epithelial Cell (AEC) proliferation	miR-627	1/61	XBP1	4/458	Regulate cell proliferation and angiogenesis
NEDD9	95/0	Cell adhesion	miR-30a	6/58	RARB	1/386	Regulate cell growth and differentiation
FLT1	94/6	Angiogenesis and vasculogenesis	miR-30e	0/45	CEBPB	1/368	Control of genes involved in immune responses
RASGEF1B	93/0	Ras guanyl-nucleotide exchange factor activity	miR-539	2/36	AR	3/363	Androgen receptor responsible for regulating gene transcription
TMEM2	88/0	Transmembrane protein	miR-495	0/34	FOXA1	0/363	Regulation of metabolism and differentiation
**Set A Genes regulatory network**							
FZD5	76/0	Receptor of Wnt signaling proteins	miR-30e	0/19	TCF4/TCF7L2	5/27	Key regulator of Wnt signaling
KCNMA1	76/0	Repolarization of membrane potential	miR-30a	6/17	VDR	0/26	Regulation of cell proliferation and calcium homeostasis
SYTL2	71/0	Vesicle trafficking	miR-30d*	0/16	ETS1	2/25	Regulation of genes belonged to development and apoptosis
SLC39A8	62/0	Transporter of Zn and toxic cadmium ion	miR-345	0/14	STAT1	0/25	Stimulation of interferons
DCLK1	60/0	Neurogenesis, neuronal migration and apoptosis	miR-539	2/13	XBP1	4/24	Regulate cell proliferation and angiogenesis

Table S12: Detailed information of high-throughput data considered for study from different Data sources

Study ID	Sample Id	Final Diagnosis	Sample Name	Tissue Type	Tissue Subtype
**GSE21411 (11 IPF, 6 Control) Gene Microarray Cho et al.**					
GSE21411	GSM533882	control	Control_1	lung	lung
GSE21411	GSM533883	control	Control_2	lung	lung
GSE21411	GSM533884	control	Control_3	lung	lung
GSE21411	GSM533885	control	Control_4	lung	lung
GSE21411	GSM533886	control	Control_5	lung	lung
GSE21411	GSM533887	control	Control_6	lung	lung
GSE21411	GSM533888	UIP/IPF	ILD_1	lung	lung
GSE21411	GSM533889	UIP/IPF	ILD_2	lung	lung
GSE21411	GSM533891	UIP/IPF	ILD_4	lung	lung
GSE21411	GSM533892	UIP/IPF	ILD_5	lung	lung
GSE21411	GSM533893	UIP/IPF	ILD_6	lung	lung
GSE21411	GSM533896	UIP/IPF	ILD_9	lung	lung
GSE21411	GSM533897	UIP/IPF	ILD_10	lung	lung
GSE21411	GSM533899	UIP/IPF	ILD_12	lung	lung
GSE21411	GSM533905	UIP/IPF	ILD_18	lung	lung
GSE21411	GSM533909	UIP/IPF	ILD_22	lung	lung
GSE21411	GSM533910	UIP/IPF	ILD_23	lung	lung
**GSE24206 (17 IPF, 6 Control) Gene Microarray Meltzer et al.**					
GSE24206	GSM595407	control	Healthy donor_biological replicate1	lung	lung
GSE24206	GSM595411	control	Healthy donor_biological replicate2	lung	lung
GSE24206	GSM595414	control	Healthy donor_biological replicate3	lung	lung
GSE24206	GSM595416	control	Healthy donor_biological replicate4	lung	lung

GSE24206	GSM595417	control	Healthy donor_biological replicate5	lung	lung
GSE24206	GSM595419	control	Healthy donor_biological replicate6	lung	lung
GSE24206	GSM595421	IPF	Early IPF_surgical biopsy_upper lobe_rep140	lung	lung, upper lobe
GSE24206	GSM595422	IPF	Early IPF_surgical biopsy_upper lobe_rep142	lung	lung, upper lobe
GSE24206	GSM595423	IPF	Early IPF_surgical biopsy_upper lobe_rep144	lung	lung, upper lobe
GSE24206	GSM595424	IPF	Early IPF_surgical biopsy_upper lobe_rep145	lung	lung, upper lobe
GSE24206	GSM595425	IPF	Advanced IPF_explant_lower lobe_rep146	lung	lung, lower lobe
GSE24206	GSM595426	IPF	Early IPF_surgical biopsy_upper lobe_rep149	lung	lung, upper lobe
GSE24206	GSM595427	IPF	Early IPF_surgical biopsy_lower lobe_rep149	lung	lung, lower lobe
GSE24206	GSM595428	IPF	Advanced IPF_explant_upper	lung	lung, upper

			lobe_rep152		lobe
GSE24206	GSM595429	IPF	Advanced IPF_explant_lower lobe_rep152	lung	lung, lower lobe
GSE24206	GSM595432	IPF	Advanced IPF_explant_upper lobe_rep157	lung	lung, upper lobe
GSE24206	GSM595434	IPF	Advanced IPF_explant_lower lobe_rep157	lung	lung, lower lobe
GSE24206	GSM595435	IPF	Advanced IPF_explant_upper lobe_rep158	lung	lung, upper lobe
GSE24206	GSM595437	IPF	Advanced IPF_explant_lower lobe_rep158	lung	lung, lower lobe
GSE24206	GSM595439	IPF	Early IPF_surgical biopsy_upper lobe_rep159	lung	lung, upper lobe
GSE24206	GSM595441	IPF	Early IPF_surgical biopsy_lower lobe_rep159	lung	lung, lower lobe
GSE24206	GSM595443	IPF	Advanced IPF_explant_upper lobe_rep160	lung	lung, upper lobe
GSE24206	GSM595445	IPF	Advanced IPF_explant_lower	lung	lung, lower

			lobe_rep160		lobe
**GSE32537 (119 IPF, 50 Control) Gene Microarray Yang et al.**					
GSE32537	GSM806204	IPF/UIP	LT000216	lung	L Lower
GSE32537	GSM806205	IPF/UIP	LT001600	lung	R Lower
GSE32537	GSM806207	IPF/UIP	LT002902	lung	R Lower
GSE32537	GSM806208	IPF/UIP	LT004173	lung	L Lower
GSE32537	GSM806209	IPF/UIP	LT006783	lung	R Lower
GSE32537	GSM806210	IPF/UIP	LT006946	lung	R Lower
GSE32537	GSM806212	IPF/UIP	LT008069	lung	R Lower
GSE32537	GSM806214	IPF/UIP	LT009429	lung	R Lower
GSE32537	GSM806216	IPF/UIP	LT012417	lung	R Lower
GSE32537	GSM806219	IPF/UIP	LT019699	lung	R Lower
GSE32537	GSM806220	IPF/UIP	LT020259	lung	L Lower
GSE32537	GSM806221	IPF/UIP	LT025724	lung	L Lower
GSE32537	GSM806222	IPF/UIP	LT026534	lung	R Lower
GSE32537	GSM806223	IPF/UIP	LT030385	lung	R Lower
GSE32537	GSM806225	IPF/UIP	LT033422	lung	R Lower
GSE32537	GSM806226	IPF/UIP	LT039091	lung	R Lower
GSE32537	GSM806227	IPF/UIP	LT041723	lung	R Lower
GSE32537	GSM806228	IPF/UIP	LT046539	lung	R Lower
GSE32537	GSM806229	IPF/UIP	LT047521	lung	R Lower
GSE32537	GSM806230	IPF/UIP	LT049193	lung	L Upper
GSE32537	GSM806231	IPF/UIP	LT056301	lung	L Upper
GSE32537	GSM806232	IPF/UIP	LT056464	lung	R Lower
GSE32537	GSM806234	IPF/UIP	LT059846	lung	R Lower
GSE32537	GSM806235	IPF/UIP	LT062141	lung	R Lower
GSE32537	GSM806236	IPF/UIP	LT062911	lung	L Upper
GSE32537	GSM806237	IPF/UIP	LT063974	lung	L Lower
GSE32537	GSM806238	IPF/UIP	LT067200	lung	L Upper
GSE32537	GSM806239	IPF/UIP	LT067836	lung	R Lower
GSE32537	GSM806240	IPF/UIP	LT069422	lung	R Lower
GSE32537	GSM806241	IPF/UIP	LT071706	lung	R Middle
GSE32537	GSM806242	IPF/UIP	LT072789	lung	R Upper
GSE32537	GSM806243	IPF/UIP	LT074868	lung	R Lower

GSE32537	GSM806247	IPF/UIP	LT080836	lung	R Middle
GSE32537	GSM806250	IPF/UIP	LT087826	lung	L Upper
GSE32537	GSM806251	IPF/UIP	LT089958	lung	R Lower
GSE32537	GSM806252	IPF/UIP	LT090283	lung	L Lower
GSE32537	GSM806253	IPF/UIP	LT091552	lung	L Lower
GSE32537	GSM806254	IPF/UIP	LT092669	lung	L Lower
GSE32537	GSM806255	IPF/UIP	LT093665	lung	L Ling
GSE32537	GSM806257	IPF/UIP	LT095103	lung	L Lower
GSE32537	GSM806258	IPF/UIP	LT098394	lung	R Lower
GSE32537	GSM806259	IPF/UIP	LT099826	lung	L Lower
GSE32537	GSM806260	IPF/UIP	LT100434	lung	L Upper
GSE32537	GSM806261	IPF/UIP	LT101536	lung	L Lower
GSE32537	GSM806262	IPF/UIP	LT102695	lung	L Lower
GSE32537	GSM806263	IPF/UIP	LT103266	lung	R Lower
GSE32537	GSM806264	IPF/UIP	LT104717	lung	R Lower
GSE32537	GSM806267	IPF/UIP	LT109321	lung	R Lower
GSE32537	GSM806268	IPF/UIP	LT112491	lung	R Middle
GSE32537	GSM806269	IPF/UIP	LT116459	lung	L Lower
GSE32537	GSM806273	IPF/UIP	LT119242	lung	R Lower
GSE32537	GSM806274	IPF/UIP	LT128191	lung	L Lower
GSE32537	GSM806275	IPF/UIP	LT128262	lung	R Upper
GSE32537	GSM806276	IPF/UIP	LT130861	lung	R Lower
GSE32537	GSM806277	IPF/UIP	LT134279	lung	L Lower
GSE32537	GSM806278	IPF/UIP	LT134302	lung	L Lower
GSE32537	GSM806279	IPF/UIP	LT134776	lung	L Lower
GSE32537	GSM806281	IPF/UIP	LT137431	lung	R Lower
GSE32537	GSM806284	IPF/UIP	LT142933	lung	R Upper
GSE32537	GSM806285	IPF/UIP	LT144921	lung	L Lower
GSE32537	GSM806286	IPF/UIP	LT148511	lung	L Upper
GSE32537	GSM806287	IPF/UIP	LT150445	lung	R Lower
GSE32537	GSM806290	IPF/UIP	LT154952	lung	R Lower
GSE32537	GSM806291	IPF/UIP	LT156276	lung	R Lower
GSE32537	GSM806292	IPF/UIP	LT157253	lung	R Lower
GSE32537	GSM806293	IPF/UIP	LT157856	lung	L Upper

GSE32537	GSM806294	IPF/UIP	LT159457	lung	L Lower
GSE32537	GSM806297	IPF/UIP	LT163953	lung	R Lower
GSE32537	GSM806298	IPF/UIP	LT165736	lung	L Lower
GSE32537	GSM806299	IPF/UIP	LT166493	lung	R Lower
GSE32537	GSM806300	IPF/UIP	LT167906	lung	L Lower
GSE32537	GSM806301	IPF/UIP	LT168219	lung	R Lower
GSE32537	GSM806302	IPF/UIP	LT168352	lung	L Lower
GSE32537	GSM806305	IPF/UIP	LT173946	lung	R Lower
GSE32537	GSM806306	IPF/UIP	LT174813	lung	L Lower
GSE32537	GSM806307	IPF/UIP	LT175399	lung	R Upper
GSE32537	GSM806308	IPF/UIP	LT180758	lung	R Middle
GSE32537	GSM806309	IPF/UIP	LT182067	lung	R Lower
GSE32537	GSM806310	IPF/UIP	LT182636	lung	R Lower
GSE32537	GSM806313	IPF/UIP	LT185396	lung	R Lower
GSE32537	GSM806314	IPF/UIP	LT187987	lung	L Lower
GSE32537	GSM806315	IPF/UIP	LT188467	lung	L Lower
GSE32537	GSM806316	IPF/UIP	LT188524	lung	R Lower
GSE32537	GSM806318	IPF/UIP	LT194091	lung	R Lower
GSE32537	GSM806320	IPF/UIP	LT195192	lung	R Lower
GSE32537	GSM806321	IPF/UIP	LT196309	lung	R Lower
GSE32537	GSM806322	IPF/UIP	LT197286	lung	L Upper
GSE32537	GSM806324	IPF/UIP	LT201348	lung	L Upper
GSE32537	GSM806327	IPF/UIP	LT205601	lung	R Lower
GSE32537	GSM806329	IPF/UIP	LT206005	lung	R Lower
GSE32537	GSM806330	IPF/UIP	LT207236	lung	R Middle
GSE32537	GSM806332	IPF/UIP	LT211379	lung	R Lower
GSE32537	GSM806333	IPF/UIP	LT211455	lung	L Upper
GSE32537	GSM806335	IPF/UIP	LT217339	lung	R Middle
GSE32537	GSM806336	IPF/UIP	LT219681	lung	R Lower
GSE32537	GSM806338	IPF/UIP	LT221381	lung	R Lower
GSE32537	GSM806339	IPF/UIP	LT228897	lung	L Lower
GSE32537	GSM806340	IPF/UIP	LT230142	lung	R Lower
GSE32537	GSM806342	IPF/UIP	LT231373	lung	L Upper
GSE32537	GSM806344	IPF/UIP	LT235441	lung	R Lower

GSE32537	GSM806346	IPF/UIP	LT242161	lung	R Lower
GSE32537	GSM806349	IPF/UIP	LT258372	lung	R Lower
GSE32537	GSM806351	IPF/UIP	LT262773	lung	L Upper
GSE32537	GSM806352	IPF/UIP	LT263234	lung	R Lower
GSE32537	GSM806354	IPF/UIP	LT264690	lung	L Upper
GSE32537	GSM806355	IPF/UIP	LT266377	lung	R Lower
GSE32537	GSM806356	IPF/UIP	LT266817	lung	L Lower
GSE32537	GSM806357	IPF/UIP	LT269381	lung	R Upper
GSE32537	GSM806358	IPF/UIP	LT269763	lung	R Lower
GSE32537	GSM806359	IPF/UIP	LT275526	lung	R Middle
GSE32537	GSM806361	IPF/UIP	LT275751	lung	R Lower
GSE32537	GSM806362	IPF/UIP	LT277811	lung	R Lower
GSE32537	GSM806363	IPF/UIP	LT280851	lung	L Lower
GSE32537	GSM806364	IPF/UIP	LT282601	lung	L Upper
GSE32537	GSM806365	IPF/UIP	LT284326	lung	R Lower
GSE32537	GSM806366	IPF/UIP	LT284489	lung	L Lower
GSE32537	GSM806368	IPF/UIP	LT287196	lung	L Lower
GSE32537	GSM806369	IPF/UIP	LT287909	lung	L Ling
GSE32537	GSM806370	IPF/UIP	LT295133	lung	L Lower
GSE32537	GSM806371	control	NJ004803	lung	Aliquot 85/RLL-P
GSE32537	GSM806372	control	NJ004804	lung	Aliquot 70/LLL-P
GSE32537	GSM806373	control	NJ004808	lung	Aliquot 91/RLL-P
GSE32537	GSM806374	control	NJ004818	lung	Aliquot 73/RLL-P
GSE32537	GSM806375	control	NJ004819	lung	Aliquot 65/LLL-P
GSE32537	GSM806376	control	NJ004824	lung	Aliquot 48/LLL-P
GSE32537	GSM806377	control	NJ004827	lung	Aliquot

					92/RLL-P
GSE32537	GSM806378	control	NJ004830	lung	Aliquot 47/RLL-P
GSE32537	GSM806379	control	NJ004840	lung	Aliquote 66/RLL-P
GSE32537	GSM806380	control	NJ004847	lung	Aliquot 41/LLL-P
GSE32537	GSM806381	control	NJ004851	lung	Aliquot 1/LLL-C
GSE32537	GSM806382	control	NJ004860	lung	Aliquot 1/RLL-P
GSE32537	GSM806383	control	NJ004863	lung	Aliquot 52/RLL-P
GSE32537	GSM806384	control	NJ020000	lung	Aliquot 6/LLL-P
GSE32537	GSM806385	control	NJ020001	lung	R Lower
GSE32537	GSM806386	control	NJ020002	lung	Aliquot 1/RLL-P
GSE32537	GSM806387	control	NJ020003	lung	Aliquot 1/RLL-P
GSE32537	GSM806388	control	NJ020005	lung	Aliquot 1/RLL-P
GSE32537	GSM806389	control	NJ020006	lung	Aliquot 1/RLL-P
GSE32537	GSM806390	control	NJ020007	lung	Aliquot 1/RLL-P
GSE32537	GSM806391	control	NJ020011	lung	Aliquot 1/RLL-P
GSE32537	GSM806392	control	NJ020015	lung	Aliquot

					7/LLL-P
GSE32537	GSM806393	control	NJ020016	lung	Aliquot 1/RLL-P
GSE32537	GSM806394	control	NJ020017	lung	Aliquot 1/RLL-P
GSE32537	GSM806395	control	NJ020022	lung	Aliquot 37/LLL
GSE32537	GSM806396	control	NJ020025	lung	Aliquot 1/LLL-P
GSE32537	GSM806397	control	NJ020026	lung	Aliquot 1/LLL-P
GSE32537	GSM806398	control	NJ020027	lung	Aliquot 67/RLL
GSE32537	GSM806399	control	NJ020028	lung	Aliquot 37/RLL-P
GSE32537	GSM806400	control	NJ020032	lung	Aliquot 62/RLL-P
GSE32537	GSM806401	control	NJ020036	lung	Aliquot 70/LLL-P
GSE32537	GSM806402	control	NJ020040	lung	Aliquot 7/RLL-C
GSE32537	GSM806403	control	NJ020042	lung	Aliquot 2/RLL-P
GSE32537	GSM806404	control	NJ020044	lung	Aliquot 2/RLL-P
GSE32537	GSM806405	control	NJ020045	lung	Aliquot 3/RLL-P
GSE32537	GSM806406	control	NJ020049	lung	Aliquot

					1/RLL-P
GSE32537	GSM806407	control	NJ020051	lung	Aliquot 74/RLL
GSE32537	GSM806408	control	NJ020053	lung	Aliquot 1/RLL-P
GSE32537	GSM806409	control	NJ020055	lung	R Lower
GSE32537	GSM806410	control	NJ020056	lung	Aliquot 1/RLL-P
GSE32537	GSM806411	control	NJ020061	lung	Aliquot 1/RLL-P
GSE32537	GSM806412	control	NJ020063	lung	Aliquot 1/RLL-P
GSE32537	GSM806413	control	NJ020064	lung	Aliquot 30/RLL
GSE32537	GSM806414	control	NJ020066	lung	Aliquot 30/RLL
GSE32537	GSM806415	control	NJ020077	lung	Aliquot 1/RLL-P
GSE32537	GSM806416	control	NJ020079	lung	Aliquot 1/RLL-C
GSE32537	GSM806417	control	NJ020080	lung	Aliquot 14/RML-P
GSE32537	GSM806418	control	NJ020081	lung	Aliquot 24/RML
GSE32537	GSM806419	control	NJ020083	lung	Aliquot 10/RML-C

GSE32537	GSM806420	control	NJ020343	lung	Aliquot 1
**GSE35147 (4 IPF, 4 Control) Gene Microarray Sanders et al.**					
GSE35147	GSM862605	IPF	RNA_IPF_2	lung	lung
GSE35147	GSM862606	IPF	RNA_IPF_3	lung	lung
GSE35147	GSM862607	IPF	RNA_IPF_C	lung	lung
GSE35147	GSM862608	IPF	RNA_IPF_S	lung	lung
GSE35147	GSM862609	control	RNA_Normal_M	lung	lung
GSE35147	GSM862610	control	RNA_Normal_N	lung	lung
GSE35147	GSM862611	control	RNA_Normal_O	lung	lung
GSE35147	GSM862612	control	RNA_Normal_W	lung	lung
**GSE10921 (4 IPF, 3 Control) Gene Microarray Vuga et al.**					
GSE10921	GSM276848	Control	NL-5	Lung	Lung
GSE10921	GSM276849	Control	NL-6	Lung	Lung
GSE10921	GSM276850	Control	NL-7	Lung	Lung
GSE10921	GSM276852	IPF/UIP	IPF-19	Lung	Lung
GSE10921	GSM276853	IPF/UIP	IPF-20	Lung	Lung
GSE10921	GSM276854	IPF/UIP	IPF-21	Lung	Lung
GSE10921	GSM276855	IPF/UIP	IPF-22	Lung	Lung
**GSE31934 (3 IPF, 3 Control) Gene Microarray Yue et al.**					
GSE31934	GSM791344	IPF	IPF3	Lung	Lung
GSE31934	GSM791345	IPF	IPF2	Lung	Lung
GSE31934	GSM791346	IPF	IPF1	Lung	Lung
GSE31934	GSM791347	Control	CON3	Lung	Lung
GSE31934	GSM791348	Control	CON2	Lung	Lung
GSE31934	GSM791349	Control	CON1	Lung	Lung
**SRP033095 (8 IPF, 7 Control) RNA-Seq Nance et al.**					
GSE52463	GSM1267256	control	Norm1	lung	lung
GSE52463	GSM1267257	control	Norm2	lung	lung
GSE52463	GSM1267258	control	Norm3	lung	lung
GSE52463	GSM1267259	control	Norm4	lung	lung
GSE52463	GSM1267260	control	Norm5	lung	lung
GSE52463	GSM1267261	control	Norm6	lung	lung
GSE52463	GSM1267262	control	Norm8	lung	lung
GSE52463	GSM1267263	IPF	IPF1	lung	lung

GSE52463	GSM1267264	IPF	IPF2	lung	lung
GSE52463	GSM1267265	IPF	IPF3	lung	lung
GSE52463	GSM1267266	IPF	IPF4	lung	lung
GSE52463	GSM1267267	IPF	IPF5	lung	lung
GSE52463	GSM1267268	IPF	IPF6	lung	lung
GSE52463	GSM1267269	IPF	IPF7	lung	lung
GSE52463	GSM1267270	IPF	IPF8	lung	lung
**SRP010041 (3 IPF, 3 Control) RNA-Seq Deng et al.**					
SRP010041	SRR393010	Control	Control1	lung	lung
SRP010041	SRR393011	Control	Control2	lung	lung
SRP010041	SRR393012	Control	Control3	lung	lung
SRP010041	SRR393013	IPF	IPF1	lung	lung
SRP010041	SRR393014	IPF	IPF2	lung	lung
SRP010041	SRR393015	IPF	IPF3	lung	lung
**GSE27430 (13 IPF, 12 Control) miRNA Microarray Milosevic et al.**					
GSE27430	GSM678128	UIP/IPF	UIP/IPF, biological rep 1	whole lung	whole lung
GSE27430	GSM678129	UIP/IPF	UIP/IPF, biological rep 2	whole lung	whole lung
GSE27430	GSM678130	UIP/IPF	UIP/IPF, biological rep 3	whole lung	whole lung
GSE27430	GSM678131	UIP/IPF	UIP/IPF, biological rep 4	whole lung	whole lung
GSE27430	GSM678132	UIP/IPF	UIP/IPF, biological rep 5	whole lung	whole lung
GSE27430	GSM678133	UIP/IPF	UIP/IPF, biological rep 6	whole lung	whole lung
GSE27430	GSM678134	UIP/IPF	UIP/IPF, biological rep 7	whole lung	whole lung
GSE27430	GSM678135	UIP/IPF	UIP/IPF, biological rep 8	whole lung	whole lung

GSE27430	GSM678136	UIP/IPF	UIP/IPF, biological rep 9	whole lung	whole lung
GSE27430	GSM678137	UIP/IPF	UIP/IPF, biological rep 10	whole lung	whole lung
GSE27430	GSM678138	UIP/IPF	UIP/IPF, biological rep 11	whole lung	whole lung
GSE27430	GSM678139	UIP/IPF	UIP/IPF, biological rep 12	whole lung	whole lung
GSE27430	GSM678140	UIP/IPF	UIP/IPF, biological rep 13	whole lung	whole lung
GSE27430	GSM678141	control	control, biological rep 1	whole lung	whole lung
GSE27430	GSM678142	control	control, biological rep 2	whole lung	whole lung
GSE27430	GSM678143	control	control, biological rep 3	whole lung	whole lung
GSE27430	GSM678144	control	control, biological rep 4	whole lung	whole lung
GSE27430	GSM678145	control	control, biological rep 5	whole lung	whole lung
GSE27430	GSM678146	control	control, biological rep 6	whole lung	whole lung
GSE27430	GSM678147	control	control, biological rep 7	whole lung	whole lung
GSE27430	GSM678148	control	control, biological rep 8	whole lung	whole lung
GSE27430	GSM678149	control	control, biological rep 9	whole lung	whole lung

GSE27430	GSM678150	control	control, biological rep 10	whole lung	whole lung
GSE27430	GSM678151	control	control, biological rep 11	whole lung	whole lung
GSE27430	GSM678152	control	control, biological rep 12	whole lung	whole lung
**GSE21394 (9 IPF, 6 Control) miRNA Microarray Cho et al.**					
GSE21394	GSM534522	UIP/IPF	ILD_1	lung	lung
GSE21394	GSM534523	UIP/IPF	ILD_2	lung	lung
GSE21394	GSM534525	UIP/IPF	ILD_5	lung	lung
GSE21394	GSM534526	UIP/IPF	ILD_6	lung	lung
GSE21394	GSM534528	UIP/IPF	ILD_9	lung	lung
GSE21394	GSM534529	UIP/IPF	ILD_10	lung	lung
GSE21394	GSM534531	UIP/IPF	ILD_12	lung	lung
GSE21394	GSM534539	UIP/IPF	ILD_22	lung	lung
GSE21394	GSM534540	UIP/IPF	ILD_23	lung	lung
GSE21394	GSM534516	Normal	Control_1	lung	lung
GSE21394	GSM534517	Normal	Control_2	lung	lung
GSE21394	GSM534518	Normal	Control_3	lung	lung
GSE21394	GSM534519	Normal	Control_4	lung	lung
GSE21394	GSM534520	Normal	Control_5	lung	lung
GSE21394	GSM534521	Normal	Control_6	lung	lung
**GSE32538 (106 IPF, 50 Control) miRNA Microarray Yang et al.**					
GSE32538	GSM806563	Control	NJ004803	Lung	Aliquot 85/RLL-P
GSE32538	GSM806564	Control	NJ004804	Lung	Aliquot 70/LLL-P
GSE32538	GSM806565	Control	NJ004808	Lung	Aliquot 91/RLL-P
GSE32538	GSM806566	Control	NJ004818	Lung	Aliquot 73/RLL-P

GSE32538	GSM806567	Control	NJ004819	Lung	Aliquot 65/LLL-P
GSE32538	GSM806568	Control	NJ004824	Lung	Aliquot 48/LLL-P
GSE32538	GSM806569	Control	NJ004827	Lung	Aliquot 92/RLL-P
GSE32538	GSM806570	Control	NJ004830	Lung	Aliquot 47/RLL-P
GSE32538	GSM806571	Control	NJ004840	Lung	Aliquote 66/RLL-P
GSE32538	GSM806572	Control	NJ004847	Lung	Aliquot 41/LLL-P
GSE32538	GSM806573	Control	NJ004851	Lung	Aliquot 1/LLL-C
GSE32538	GSM806574	Control	NJ004860	Lung	Aliquot 1/RLL-P
GSE32538	GSM806575	Control	NJ004863	Lung	Aliquot 52/RLL-P
GSE32538	GSM806576	Control	NJ020000	Lung	Aliquot 6/LLL-P
GSE32538	GSM806577	Control	NJ020001	Lung	R Lower
GSE32538	GSM806578	Control	NJ020002	Lung	Aliquot 1/RLL-P
GSE32538	GSM806579	Control	NJ020003	Lung	Aliquot 1/RLL-P
GSE32538	GSM806580	Control	NJ020005	Lung	Aliquot 1/RLL-P
GSE32538	GSM806581	Control	NJ020006	Lung	Aliquot

					1/RLL-P
GSE32538	GSM806582	Control	NJ020007	Lung	Aliquot 1/RLL-P
GSE32538	GSM806583	Control	NJ020011	Lung	Aliquot 1/RLL-P
GSE32538	GSM806584	Control	NJ020012	Lung	Aliquot 6/LLL-P
GSE32538	GSM806585	Control	NJ020015	Lung	Aliquot 7/LLL-P
GSE32538	GSM806586	Control	NJ020016	Lung	Aliquot 1/RLL-P
GSE32538	GSM806587	Control	NJ020022	Lung	Aliquot 37/LLL
GSE32538	GSM806588	Control	NJ020025	Lung	Aliquot 1/LLL-P
GSE32538	GSM806589	Control	NJ020026	Lung	Aliquot 1/LLL-P
GSE32538	GSM806590	Control	NJ020027	Lung	Aliquot 67/RLL
GSE32538	GSM806591	Control	NJ020028	Lung	Aliquot 37/RLL-P
GSE32538	GSM806592	Control	NJ020032	Lung	Aliquot 62/RLL-P
GSE32538	GSM806593	Control	NJ020036	Lung	Aliquot 70/LLL-P
GSE32538	GSM806594	Control	NJ020040	Lung	Aliquot 7/RLL-C
GSE32538	GSM806595	Control	NJ020042	Lung	Aliquot

					2/RLL-P
GSE32538	GSM806596	Control	NJ020044	Lung	Aliquot 2/RLL-P
GSE32538	GSM806597	Control	NJ020045	Lung	Aliquot 3/RLL-P
GSE32538	GSM806598	Control	NJ020049	Lung	Aliquot 1/RLL-P
GSE32538	GSM806599	Control	NJ020051	Lung	Aliquot 74/RLL
GSE32538	GSM806600	Control	NJ020053	Lung	Aliquot 1/RLL-P
GSE32538	GSM806601	Control	NJ020055	Lung	R Lower
GSE32538	GSM806602	Control	NJ020056	Lung	Aliquot 1/RLL-P
GSE32538	GSM806603	Control	NJ020061	Lung	Aliquot 1/RLL-P
GSE32538	GSM806604	Control	NJ020063	Lung	Aliquot 1/RLL-P
GSE32538	GSM806605	Control	NJ020064	Lung	Aliquot 30/RLL
GSE32538	GSM806606	Control	NJ020065	Lung	Aliquot 1/LLL-P
GSE32538	GSM806607	Control	NJ020066	Lung	Aliquot 30/RLL
GSE32538	GSM806608	Control	NJ020079	Lung	Aliquot 1/RLL-C
GSE32538	GSM806609	Control	NJ020080	Lung	Aliquot 14/RML-

					P
GSE32538	GSM806610	Control	NJ020081	Lung	Aliquot 24/RML
GSE32538	GSM806611	Control	NJ020083	Lung	Aliquot 10/RML-C
GSE32538	GSM806612	Control	NJ020343	Lung	Aliquot 1
GSE32538	GSM806421	IPF/UIP	LT000216	Lung	L Lower
GSE32538	GSM806422	IPF/UIP	LT001600	Lung	R Lower
GSE32538	GSM806424	IPF/UIP	LT002902	Lung	R Lower
GSE32538	GSM806425	IPF/UIP	LT004173	Lung	L Lower
GSE32538	GSM806426	IPF/UIP	LT006783	Lung	R Lower
GSE32538	GSM806427	IPF/UIP	LT006946	Lung	R Lower
GSE32538	GSM806429	IPF/UIP	LT008069	Lung	R Lower
GSE32538	GSM806431	IPF/UIP	LT009429	Lung	R Lower
GSE32538	GSM806433	IPF/UIP	LT019699	Lung	R Lower
GSE32538	GSM806434	IPF/UIP	LT025724	Lung	L Lower
GSE32538	GSM806435	IPF/UIP	LT030385	Lung	R Lower
GSE32538	GSM806437	IPF/UIP	LT033422	Lung	R Lower
GSE32538	GSM806438	IPF/UIP	LT039091	Lung	R Lower
GSE32538	GSM806439	IPF/UIP	LT046539	Lung	R Lower
GSE32538	GSM806440	IPF/UIP	LT047521	Lung	R Lower
GSE32538	GSM806441	IPF/UIP	LT056301	Lung	L Upper
GSE32538	GSM806442	IPF/UIP	LT056464	Lung	R Lower
GSE32538	GSM806444	IPF/UIP	LT059846	Lung	R Lower
GSE32538	GSM806445	IPF/UIP	LT062796	Lung	L Lower
GSE32538	GSM806446	IPF/UIP	LT062911	Lung	L Upper
GSE32538	GSM806447	IPF/UIP	LT063974	Lung	L Lower
GSE32538	GSM806448	IPF/UIP	LT067200	Lung	L Upper
GSE32538	GSM806449	IPF/UIP	LT067836	Lung	R Lower
GSE32538	GSM806450	IPF/UIP	LT069422	Lung	R Lower
GSE32538	GSM806451	IPF/UIP	LT071706	Lung	R Middle
GSE32538	GSM806452	IPF/UIP	LT072789	Lung	R Upper

GSE32538	GSM806453	IPF/UIP	LT074868	Lung	R Lower
GSE32538	GSM806457	IPF/UIP	LT080836	Lung	R Middle
GSE32538	GSM806460	IPF/UIP	LT087826	Lung	L Upper
GSE32538	GSM806461	IPF/UIP	LT089958	Lung	R Lower
GSE32538	GSM806462	IPF/UIP	LT090283	Lung	L Lower
GSE32538	GSM806463	IPF/UIP	LT091552	Lung	L Lower
GSE32538	GSM806464	IPF/UIP	LT092669	Lung	L Lower
GSE32538	GSM806465	IPF/UIP	LT093665	Lung	L Ling
GSE32538	GSM806467	IPF/UIP	LT095103	Lung	L Lower
GSE32538	GSM806468	IPF/UIP	LT098394	Lung	R Lower
GSE32538	GSM806469	IPF/UIP	LT099826	Lung	L Lower
GSE32538	GSM806470	IPF/UIP	LT100434	Lung	L Upper
GSE32538	GSM806471	IPF/UIP	LT100821	Lung	R Lower
GSE32538	GSM806472	IPF/UIP	LT101536	Lung	L Lower
GSE32538	GSM806473	IPF/UIP	LT103266	Lung	R Lower
GSE32538	GSM806474	IPF/UIP	LT104717	Lung	R Lower
GSE32538	GSM806477	IPF/UIP	LT109321	Lung	R Lower
GSE32538	GSM806478	IPF/UIP	LT116459	Lung	L Lower
GSE32538	GSM806481	IPF/UIP	LT119242	Lung	R Lower
GSE32538	GSM806482	IPF/UIP	LT128191	Lung	L Lower
GSE32538	GSM806483	IPF/UIP	LT128262	Lung	R Upper
GSE32538	GSM806484	IPF/UIP	LT134279	Lung	L Lower
GSE32538	GSM806485	IPF/UIP	LT134302	Lung	L Lower
GSE32538	GSM806486	IPF/UIP	LT134776	Lung	L Lower
GSE32538	GSM806488	IPF/UIP	LT137431	Lung	R Lower
GSE32538	GSM806490	IPF/UIP	LT144921	Lung	L Lower
GSE32538	GSM806491	IPF/UIP	LT150445	Lung	R Lower
GSE32538	GSM806493	IPF/UIP	LT154952	Lung	R Lower
GSE32538	GSM806494	IPF/UIP	LT156276	Lung	R Lower
GSE32538	GSM806495	IPF/UIP	LT157253	Lung	R Lower
GSE32538	GSM806496	IPF/UIP	LT159457	Lung	L Lower
GSE32538	GSM806498	IPF/UIP	LT163953	Lung	R Lower
GSE32538	GSM806499	IPF/UIP	LT165736	Lung	L Lower
GSE32538	GSM806500	IPF/UIP	LT167906	Lung	L Lower

GSE32538	GSM806501	IPF/UIP	LT168219	Lung	R Lower
GSE32538	GSM806502	IPF/UIP	LT168352	Lung	L Lower
GSE32538	GSM806503	IPF/UIP	LT173946	Lung	R Lower
GSE32538	GSM806504	IPF/UIP	LT175399	Lung	R Upper
GSE32538	GSM806505	IPF/UIP	LT180758	Lung	R Middle
GSE32538	GSM806506	IPF/UIP	LT182067	Lung	R Lower
GSE32538	GSM806507	IPF/UIP	LT182636	Lung	R Lower
GSE32538	GSM806510	IPF/UIP	LT185396	Lung	R Lower
GSE32538	GSM806511	IPF/UIP	LT187987	Lung	L Lower
GSE32538	GSM806512	IPF/UIP	LT188467	Lung	L Lower
GSE32538	GSM806513	IPF/UIP	LT188524	Lung	R Lower
GSE32538	GSM806515	IPF/UIP	LT194091	Lung	R Lower
GSE32538	GSM806517	IPF/UIP	LT195192	Lung	R Lower
GSE32538	GSM806518	IPF/UIP	LT196309	Lung	R Lower
GSE32538	GSM806519	IPF/UIP	LT197286	Lung	L Upper
GSE32538	GSM806520	IPF/UIP	LT198096	Lung	L Lower
GSE32538	GSM806521	IPF/UIP	LT201348	Lung	L Upper
GSE32538	GSM806524	IPF/UIP	LT205601	Lung	R Lower
GSE32538	GSM806526	IPF/UIP	LT206005	Lung	R Lower
GSE32538	GSM806527	IPF/UIP	LT207236	Lung	R Middle
GSE32538	GSM806529	IPF/UIP	LT211379	Lung	R Lower
GSE32538	GSM806530	IPF/UIP	LT211455	Lung	L Upper
GSE32538	GSM806532	IPF/UIP	LT217339	Lung	R Middle
GSE32538	GSM806533	IPF/UIP	LT219681	Lung	R Lower
GSE32538	GSM806534	IPF/UIP	LT221381	Lung	R Lower
GSE32538	GSM806535	IPF/UIP	LT228897	Lung	L Lower
GSE32538	GSM806536	IPF/UIP	LT230142	Lung	R Lower
GSE32538	GSM806538	IPF/UIP	LT231373	Lung	L Upper
GSE32538	GSM806539	IPF/UIP	LT235441	Lung	R Lower
GSE32538	GSM806541	IPF/UIP	LT242161	Lung	R Lower
GSE32538	GSM806542	IPF/UIP	LT244824	Lung	R Lower
GSE32538	GSM806544	IPF/UIP	LT258372	Lung	R Lower
GSE32538	GSM806546	IPF/UIP	LT263234	Lung	R Lower
GSE32538	GSM806548	IPF/UIP	LT264690	Lung	L Upper

GSE32538	GSM806549	IPF/UIP	LT266377	Lung	R Lower
GSE32538	GSM806550	IPF/UIP	LT266817	Lung	L Lower
GSE32538	GSM806551	IPF/UIP	LT269381	Lung	R Upper
GSE32538	GSM806552	IPF/UIP	LT269763	Lung	R Lower
GSE32538	GSM806554	IPF/UIP	LT275751	Lung	R Lower
GSE32538	GSM806555	IPF/UIP	LT280851	Lung	L Lower
GSE32538	GSM806556	IPF/UIP	LT282601	Lung	L Upper
GSE32538	GSM806557	IPF/UIP	LT284326	Lung	R Lower
GSE32538	GSM806558	IPF/UIP	LT284489	Lung	L Lower
GSE32538	GSM806560	IPF/UIP	LT287909	Lung	L Ling
GSE32538	GSM806561	IPF/UIP	LT295133	Lung	L Lower
GSE32538	GSM806562	IPF/UIP	LT298606	Lung	R Middle
**GSE16538 (6 Sarcoidosis, 6 Control) Gene Microarray Crouser et al.**					
GSE16538	GSM415386	Control	Lung-normal-rep1	Lung	Lung
GSE16538	GSM415387	Control	Lung-normal-rep2	Lung	Lung
GSE16538	GSM415388	Control	Lung-normal-rep3	Lung	Lung
GSE16538	GSM415389	Control	Lung-normal-rep4	Lung	Lung
GSE16538	GSM415390	Control	Lung-normal-rep5	Lung	Lung
GSE16538	GSM415391	Control	Lung-normal-rep6	Lung	Lung
GSE16538	GSM415392	Sarcoidosis	Lung-Sarcoidosis-rep1	Lung	Lung
GSE16538	GSM415393	Sarcoidosis	Lung-Sarcoidosis-rep2	Lung	Lung
GSE16538	GSM415394	Sarcoidosis	Lung-Sarcoidosis-rep3	Lung	Lung
GSE16538	GSM415395	Sarcoidosis	Lung-Sarcoidosis-rep4	Lung	Lung
GSE16538	GSM415396	Sarcoidosis	Lung-Sarcoidosis-rep5	Lung	Lung
GSE16538	GSM415397	Sarcoidosis	Lung-Sarcoidosis-rep6	Lung	Lung
**GSE19976 (15 Sarcoidosis) Gene Microarray Lockstone et al.**					
GSE19976	GSM499117	Sarcoidosis	Lung_self-limiting_biological_rep1	Lung	Lung
GSE19976	GSM499118	Sarcoidosis	Lung_self-limiting_biological_rep2	Lung	Lung
GSE19976	GSM499119	Sarcoidosis	Lung_self-limiting_biological_rep3	Lung	Lung
GSE19976	GSM499120	Sarcoidosis	Lung_self-	Lung	Lung

			limiting_biological_rep4		
GSE19976	GSM499121	Sarcoidosis	Lung_self-limiting_biological_rep5	Lung	Lung
GSE19976	GSM499122	Sarcoidosis	Lung_self-limiting_biological_rep6	Lung	Lung
GSE19976	GSM499123	Sarcoidosis	Lung_self-limiting_biological_rep7	Lung	Lung
GSE19976	GSM499124	Sarcoidosis	Lung_self-limiting_biological_rep8	Lung	Lung
GSE19976	GSM499125	Sarcoidosis	Lung_Progressive_biological_rep1	Lung	Lung
GSE19976	GSM499126	Sarcoidosis	Lung_Progressive_biological_rep2	Lung	Lung
GSE19976	GSM499127	Sarcoidosis	Lung_Progressive_biological_rep3	Lung	Lung
GSE19976	GSM499128	Sarcoidosis	Lung_Progressive_biological_rep4	Lung	Lung
GSE19976	GSM499129	Sarcoidosis	Lung_Progressive_biological_rep5	Lung	Lung
GSE19976	GSM499130	Sarcoidosis	Lung_Progressive_biological_rep6	Lung	Lung
GSE19976	GSM499131	Sarcoidosis	Lung_Progressive_biological_rep7	Lung	Lung
**GSE5774 (12 NSIP) Gene Microarray Yang et al.**					
GSE5774	GSM133542	NSIP	F-IIP(NSIP)1_Cy3	Lung	Lung
GSE5774	GSM133544	NSIP	F-IIP(NSIP)1_Cy5	Lung	Lung
GSE5774	GSM133547	NSIP	F-IIP(NSIP)2_Cy3	Lung	Lung
GSE5774	GSM133548	NSIP	F-IIP(NSIP)2_Cy5	Lung	Lung
GSE5774	GSM133674	NSIP	S-IIP(NSIP)1_Cy3	Lung	Lung
GSE5774	GSM133675	NSIP	S-IIP(NSIP)1_Cy5	Lung	Lung

GSE5774	GSM133680	NSIP	F-IIP(NSIP)3_Cy3	Lung	Lung
GSE5774	GSM133682	NSIP	F-IIP(NSIP)3_Cy5	Lung	Lung
GSE5774	GSM134673	NSIP	S-IIP(NSIP)10_Cy3	Lung	Lung
GSE5774	GSM134674	NSIP	S-IIP(NSIP)10_Cy5	Lung	Lung
GSE5774	GSM134675	NSIP	F-IIP(NSIP)5_Cy3	Lung	Lung
GSE5774	GSM134676	NSIP	F-IIP(NSIP)5_Cy5	Lung	Lung

References:

1. Sing, T., Sander, O., Beerenwinkel, N. & Lengauer, T. ROCR: visualizing classifier performance in R. Bioinformatics 21, 3940–3941 (2005).
2. Yoshioka, H. et al. In vivo therapeutic effect of CDH3/P-cadherin-targeting radioimmunotherapy. Cancer Immunol. Immunother. 61, 1211–1220 (2012).
3. He, W. et al. Exogenously administered secreted frizzled related protein 2 (Sfrp2) reduces fibrosis and improves cardiac function in a rat model of myocardial infarction. Proc. Natl. Acad. Sci. U.S.A. 107, 21110–21115 (2010).
4. Vuga, L. J. et al. WNT5A Is a Regulator of Fibroblast Proliferation and Resistance to Apoptosis. Am J Respir Cell Mol Biol 41, 583–589 (2009).
5. Burkhardt, A. M. et al. CXCL17 Is a Mucosal Chemokine Elevated in Idiopathic Pulmonary Fibrosis That Exhibits Broad Antimicrobial Activity. J Immunol 188, 6399–6406 (2012).
6. P, C. & J, P. Regulatory Roles of Dclk1 in Epithelial Mesenchymal Transition and Cancer

Stem Cells. Journal of Carcinogenesis & Mutagenesis 7, (2016).

7. Guoying Yu et al. in C68. OLD AND NEW PATHWAYS TO PULMONARY FIBROSIS A4932–A4932 (American Thoracic Society, 2012).
8. Bauer, Y. et al. A novel genomic signature with translational significance for human idiopathic pulmonary fibrosis. Am. J. Respir. Cell Mol. Biol. 52, 217–231 (2015).
9. Arderiu, G., Espinosa, S., Peña, E., Aledo, R. & Badimon, L. Monocyte-secreted Wnt5a interacts with FZD5 in microvascular endothelial cells and induces angiogenesis through tissue factor signaling. J Mol Cell Biol 6, 380–393 (2014).
10. Leppäranta, O. et al. Regulation of TGF-β storage and activation in the human idiopathic pulmonary fibrosis lung. Cell Tissue Res. 348, 491–503 (2012)
11. Plantier, L. et al. Ectopic respiratory epithelial cell differentiation in bronchiolised distal airspaces in idiopathic pulmonary fibrosis. Thorax 66, 651–657 (2011).
12. Vervoort, S. J., Lourenço, A. R., van Boxtel, R. & Coffer, P. J. SOX4 mediates TGF-β-induced expression of mesenchymal markers during mammary cell epithelial to mesenchymal transition. PLoS ONE 8, e53238 (2013).
13. Győry, I. et al. Transcription factor Ebf1 regulates differentiation stage-specific signaling, proliferation, and survival of B cells. Genes Dev. 26, 668–682 (2012).
14. Micalizzi, D. S. et al. The Six1 homeoprotein induces human mammary carcinoma cells to undergo epithelial-mesenchymal transition and metastasis in mice through increasing TGF-beta signaling. J. Clin. Invest. 119, 2678–2690 (2009).
15. Liu, Y., Chu, A., Chakroun, I., Islam, U. & Blais, A. Cooperation between myogenic regulatory factors and SIX family transcription factors is important for myoblast differentiation. Nucleic Acids Res 38, 6857–6871 (2010).
16. Koinuma, D. *et al.* Chromatin immunoprecipitation on microarray analysis of Smad2/3

binding sites reveals roles of ETS1 and TFAP2A in transforming growth factor beta signaling. *Mol. Cell. Biol.* **29,** 172–186 (2009).

17. Luchsinger, L. L., Patenaude, C. A., Smith, B. D. & Layne, M. D. Myocardin-related transcription factor-A complexes activate type I collagen expression in lung fibroblasts. J. Biol. Chem. 286, 44116–44125 (2011).
18. Murata, K. et al. p63 - Key molecule in the early phase of epithelial abnormality in idiopathic pulmonary fibrosis. Exp. Mol. Pathol. 83, 367–376 (2007).
19. Sho, T. et al. TRIM29 negatively regulates p53 via inhibition of Tip60. Biochim. Biophys. Acta 1813, 1245–1253 (2011).
20. Lee, K. S., Buck, M., Houglum, K. & Chojkier, M. Activation of hepatic stellate cells by TGF alpha and collagen type I is mediated by oxidative stress through c-myb expression. J. Clin. Invest. 96, 2461–2468 (1995).
21. Yan, C. *et al.* CCAAT/enhancer-binding protein δ is a critical mediator of lipopolysaccharide-induced acute lung injury. *Am. J. Pathol.* **182,** 420–430 (2013).
22. Nakamura, T. et al. Axin, an inhibitor of the Wnt signalling pathway, interacts with beta-catenin, GSK-3beta and APC and reduces the beta-catenin level. Genes Cells 3, 395–403 (1998).
23. Königshoff, M. et al. Functional Wnt signaling is increased in idiopathic pulmonary fibrosis. PLoS ONE 3, e2142 (2008).
24. Rajasekaran, S., Vaz, M. & Reddy, S. P. Fra-1/AP-1 transcription factor negatively regulates pulmonary fibrosis in vivo. PLoS ONE 7, e41611 (2012).
25. Li, Q. F., Wang, X. R., Yang, Y. W. & Lin, H. Hypoxia upregulates hypoxia inducible factor (HIF)-3alpha expression in lung epithelial cells: characterization and comparison with HIF-1alpha. Cell Res. 16, 548–558 (2006).

26. Yin, Z. et al. Hop functions downstream of Nkx2.1 and GATA6 to mediate HDAC-dependent negative regulation of pulmonary gene expression. Am. J. Physiol. Lung Cell Mol. Physiol. 291, L191-199 (2006).
27. Myllärniemi, M. et al. Gremlin-mediated decrease in bone morphogenetic protein signaling promotes pulmonary fibrosis. Am. J. Respir. Crit. Care Med. 177, 321–329 (2008).
28. Patel, N. M. et al. Pulmonary arteriole gene expression signature in idiopathic pulmonary fibrosis. Eur. Respir. J. 41, 1324–1330 (2013).
29. Lu, Y. et al. Kruppel-like factor 15 regulates smooth muscle response to vascular injury--brief report. Arterioscler. Thromb. Vasc. Biol. 30, 1550–1552 (2010).
30. Fujita, R. *et al.* NF-E2 p45 is important for establishing normal function of platelets. *Mol. Cell. Biol.* **33,** 2659–2670 (2013).
31. Selman, M. *et al.* Accelerated Variant of Idiopathic Pulmonary Fibrosis: Clinical Behavior and Gene Expression Pattern. *PLoS ONE* **2,** (2007).
32. Singh, D. *et al.* Altered gene expression in blood and sputum in COPD frequent exacerbators in the ECLIPSE cohort. *PLoS ONE* **9,** e107381 (2014).
33. Kuwano, K. *et al.* P21Waf1/Cip1/Sdi1 and p53 expression in association with DNA strand breaks in idiopathic pulmonary fibrosis. *Am. J. Respir. Crit. Care Med.* 154, 477–483 (1996).
34. Sunaga, H. *et al.* Deranged fatty acid composition causes pulmonary fibrosis in Elovl6-deficient mice. *Nat Commun* 4, 2563 (2013).

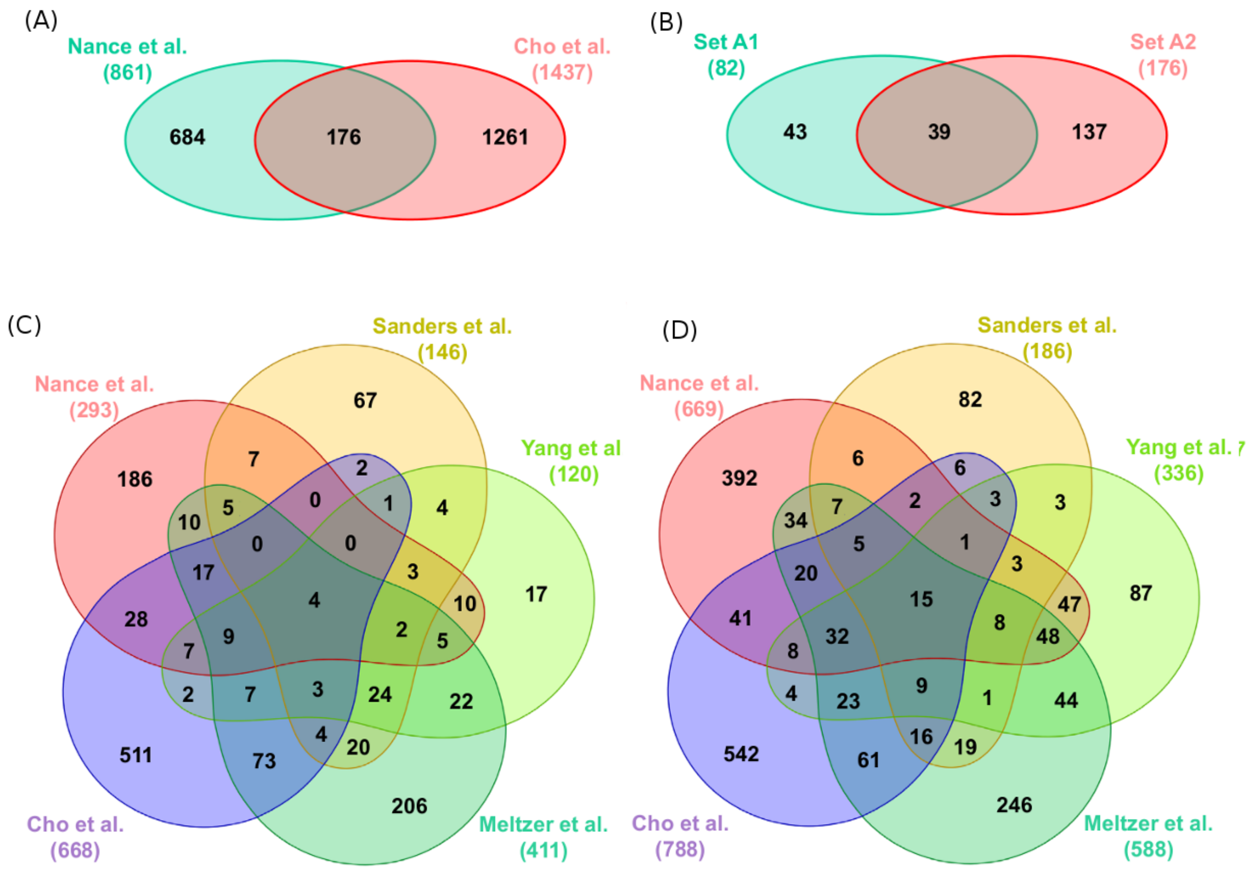

Figure S1: Overlap of DEGs among different experiments **(A)** SRP033095 and GSE21411, **(B)** previously reported 82 genes and 176 genes from our study, **(C)** Down regulated genes of GSE35147, GSE32537, GSE21411, GSE24206 and SRP033095, **(D)** Up regulated genes of GSE35147, GSE32537, GSE21411, GSE24206 and SRP033095

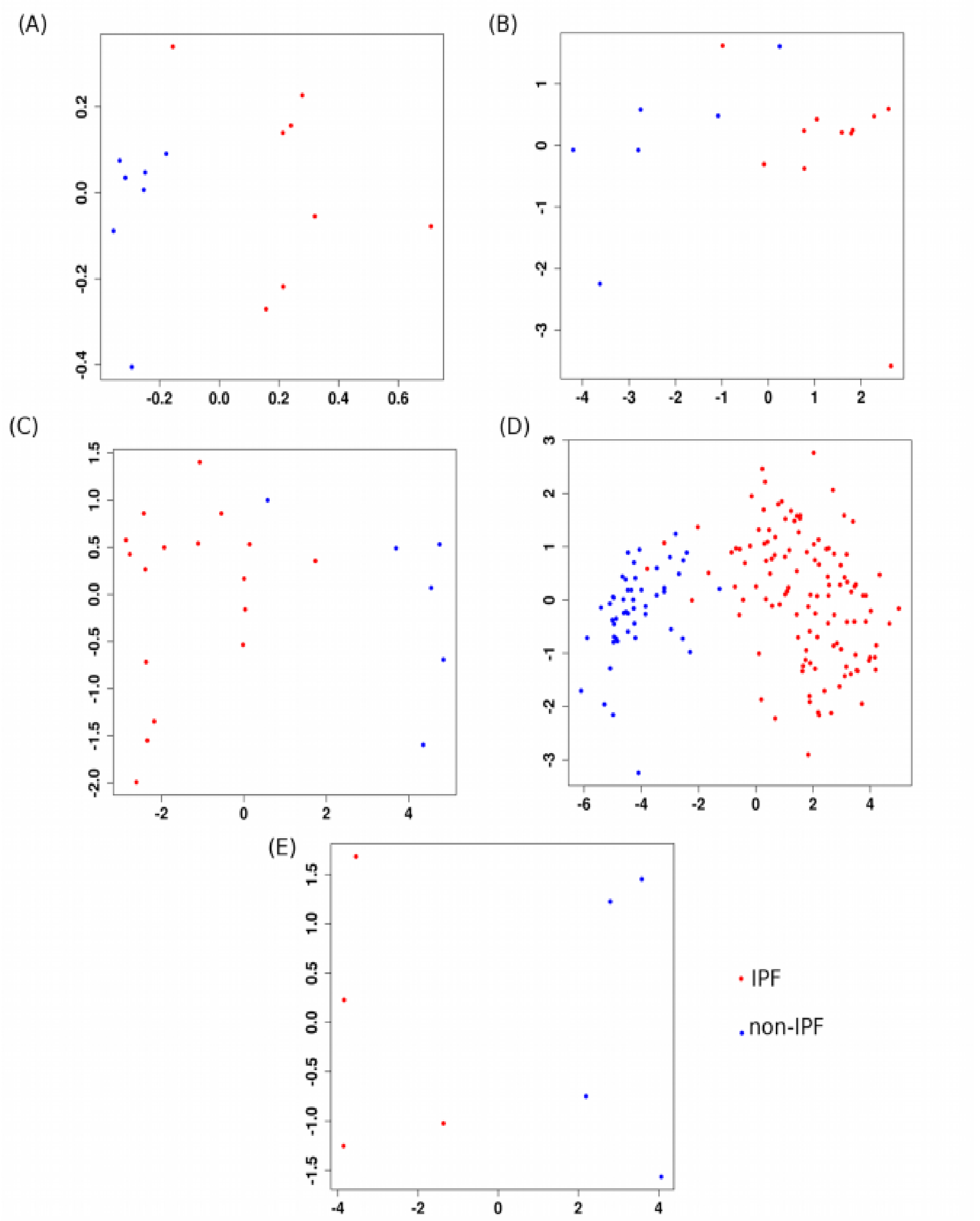

Figure S2: Clustering results of Set A1 for different experiments **(A)** SRP033095, **(B)** GSE21411, **(C)** GSE24206, **(D)** GSE32537, **(E)** GSE35147

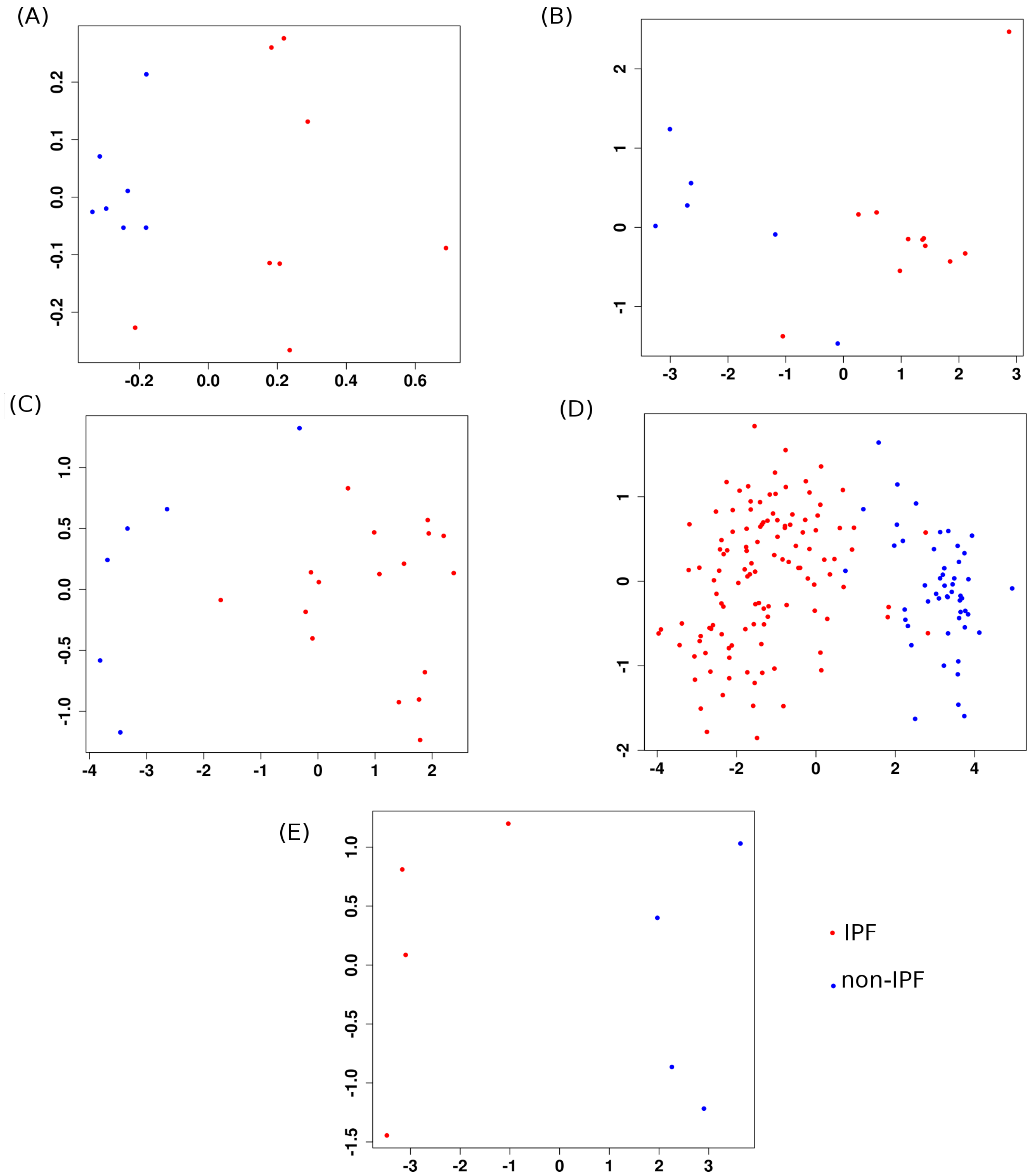

Figure S3: Clustering results of Set A for different experiments **(A)** SRP033095, **(B)** GSE21411, **(C)** GSE24206, **(D)** GSE32537, **(E)** GSE35147

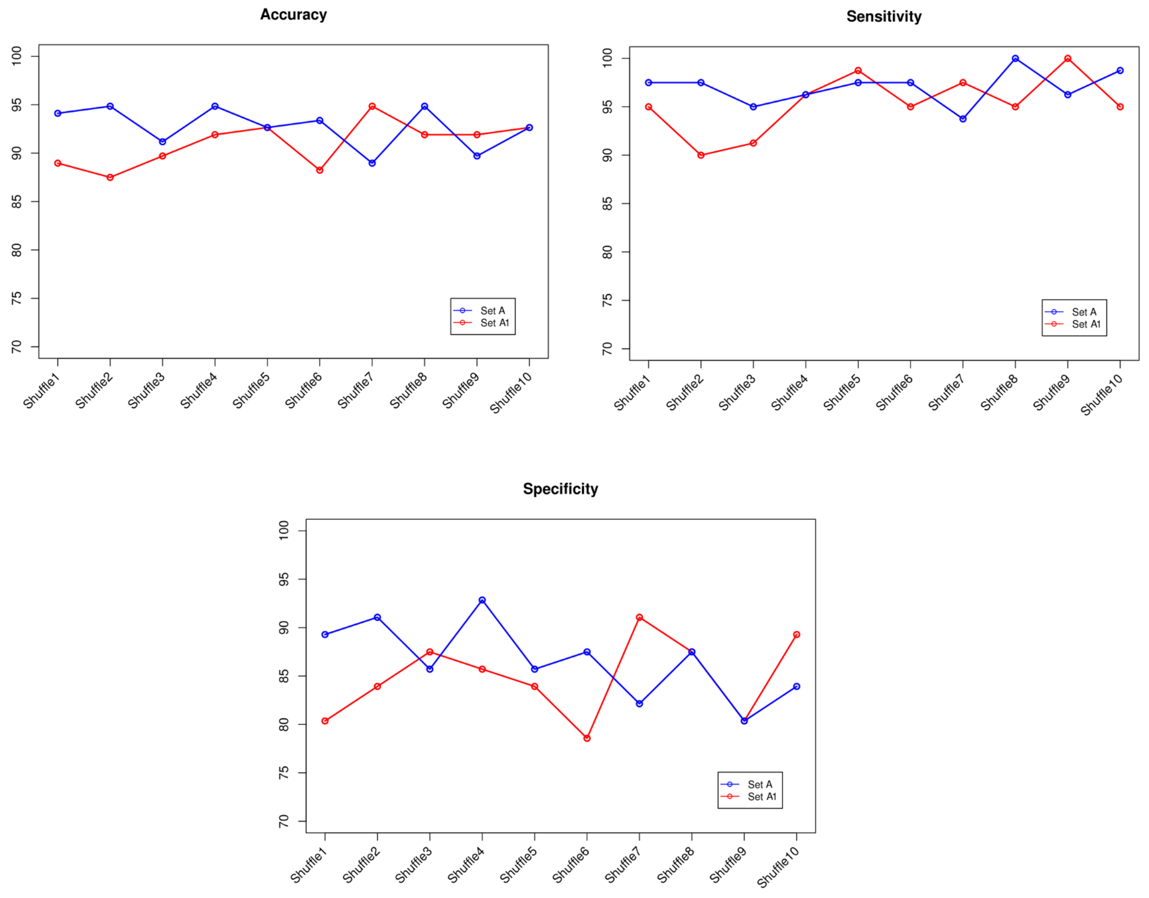

Figure S4: Accuracy, Sensitivity and Specificity plot for Set A and Set A1 on 10 randomized dataset models

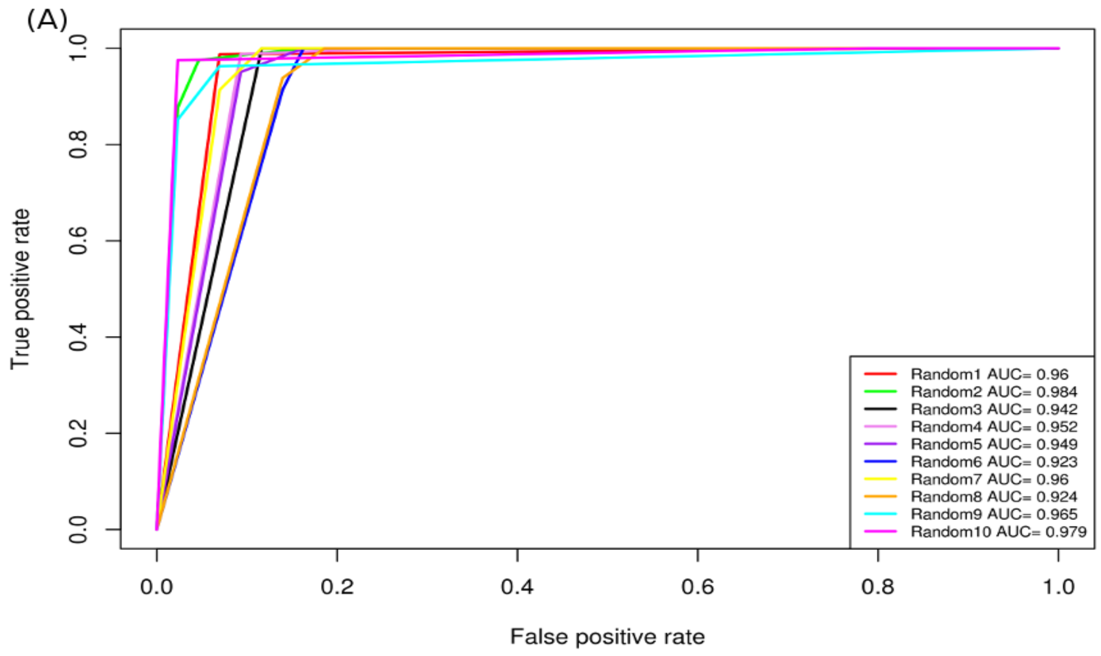

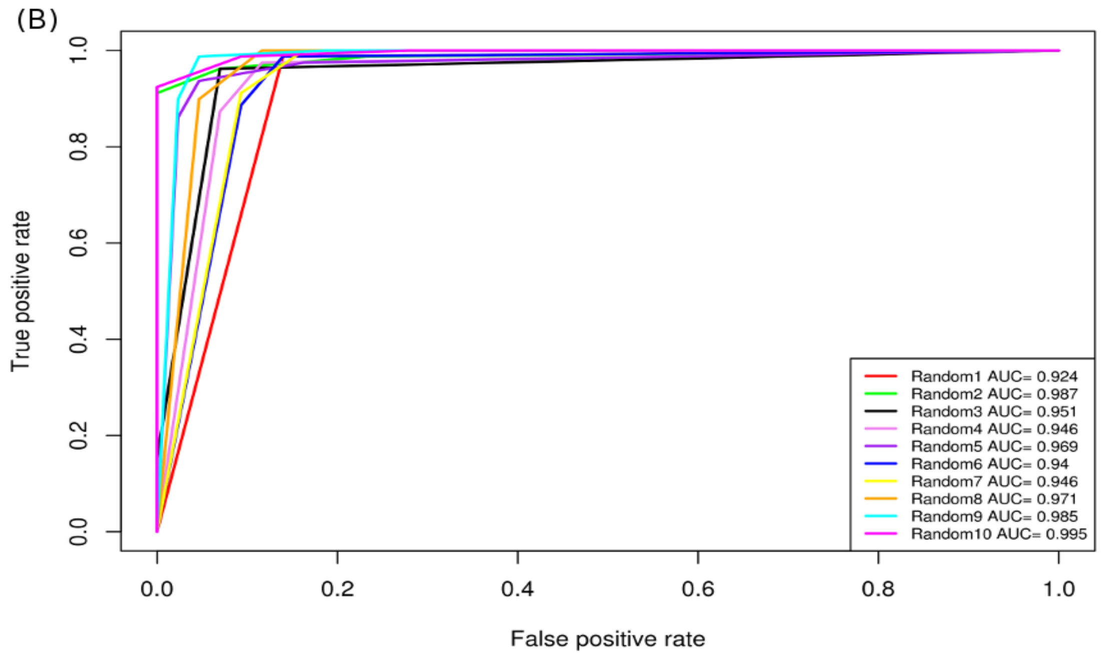

Figure S5: ROC plots of two gene sets against ten random models **(A)** Set A **(B)** Set A1

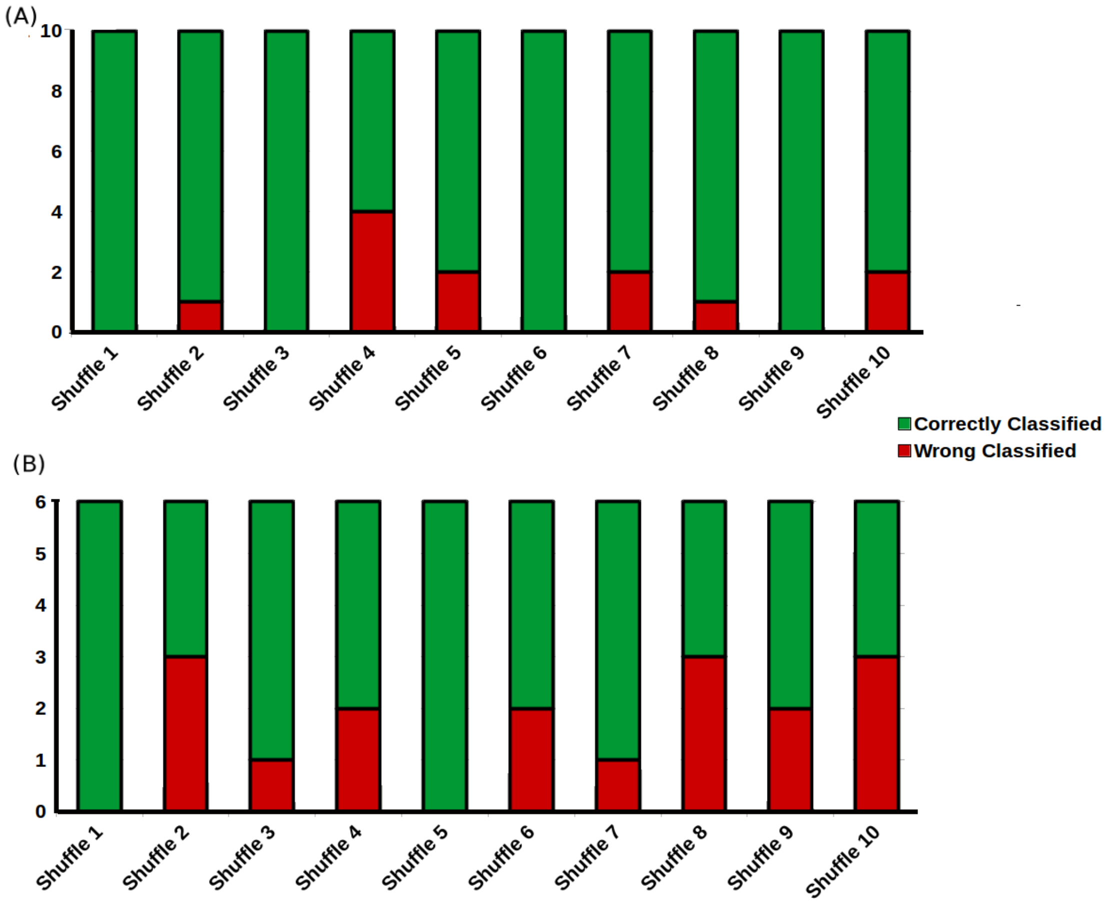

Figure S6: NSIP and Sarcoidosis individuals classification on 10 different models of Set A **(A)** Sarcoidosis (10 Individuals) **(B)** NSIP (6 Individuals)

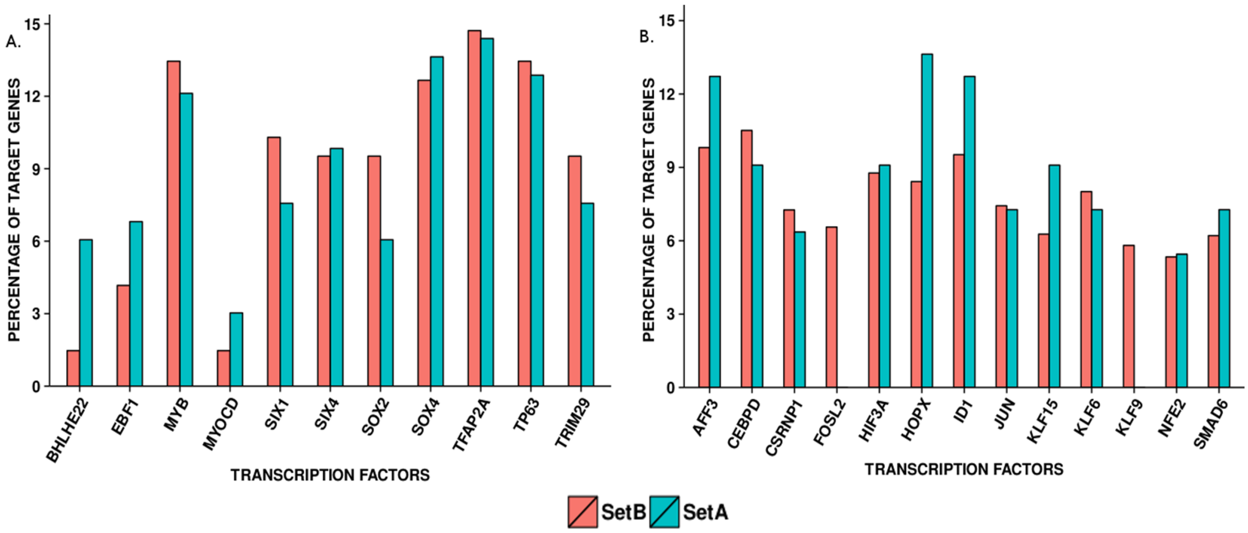

Figure S7: (A) Ranking of differentially expressed upregulated transcription factors regulating DEGs, **(B)** Ranking of differentially expressed downregulated transcription factors regulating DEGs specific to IPF

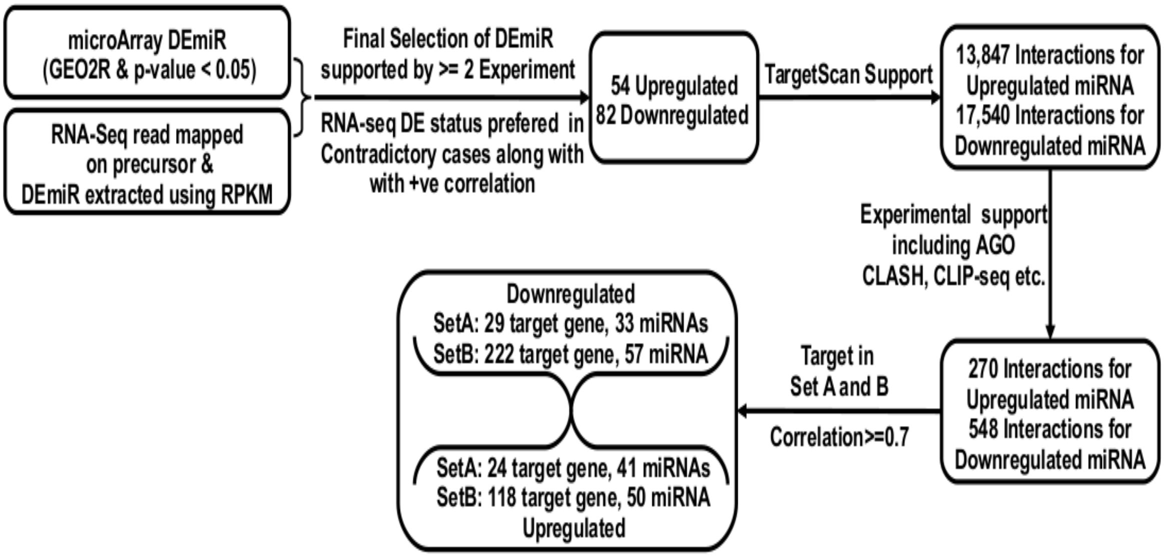

Figure S8: Workflow of miRNA analysis from microarray and RNA-seq study, miRNA target finding, support analysis and involvement of miRNA in different sets of genes

(A)

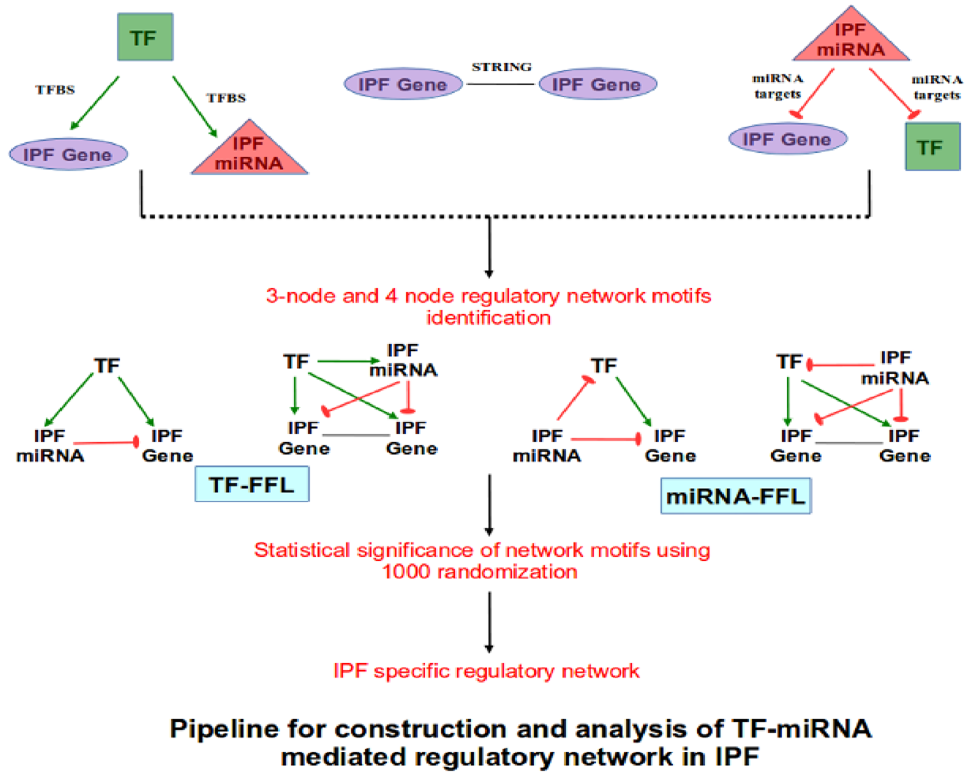

(B)

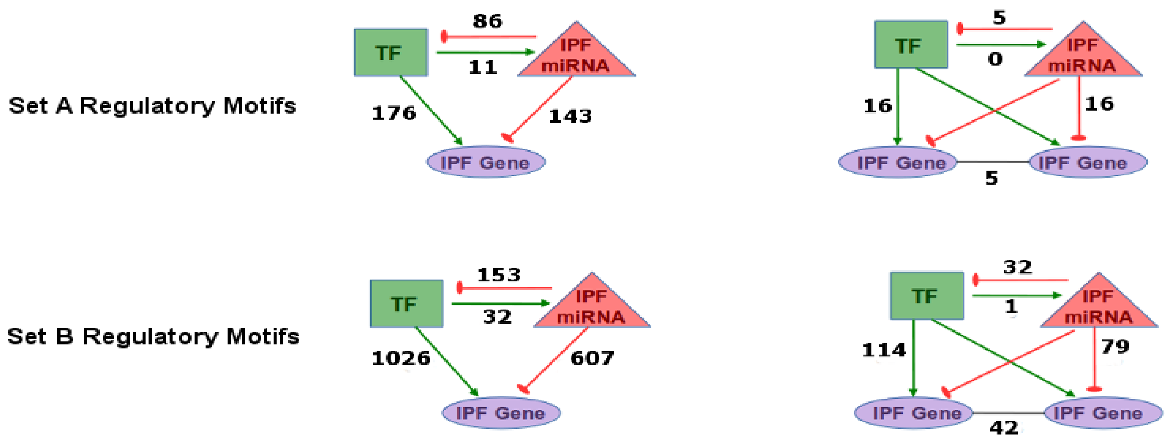

Figure S9: (A) Workflow for generation of various potential Feed-Forward loops and construction of TF-miRNA mediated regulatory network in IPF, **(B)** Statistics of three and four node potential FFLs for the three sets of DEGs in IPF.

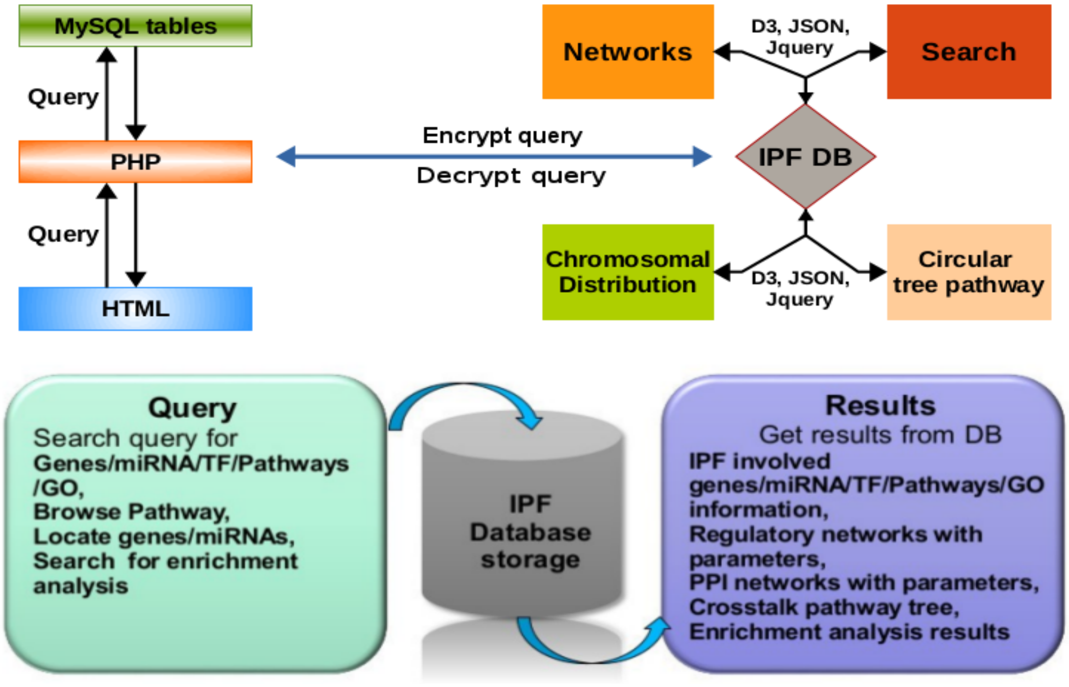

Figure S10: The framework and workflow of IPF portal. User input query is processed in background through PHP and MySQL and retrieved results are visualized in HTML.

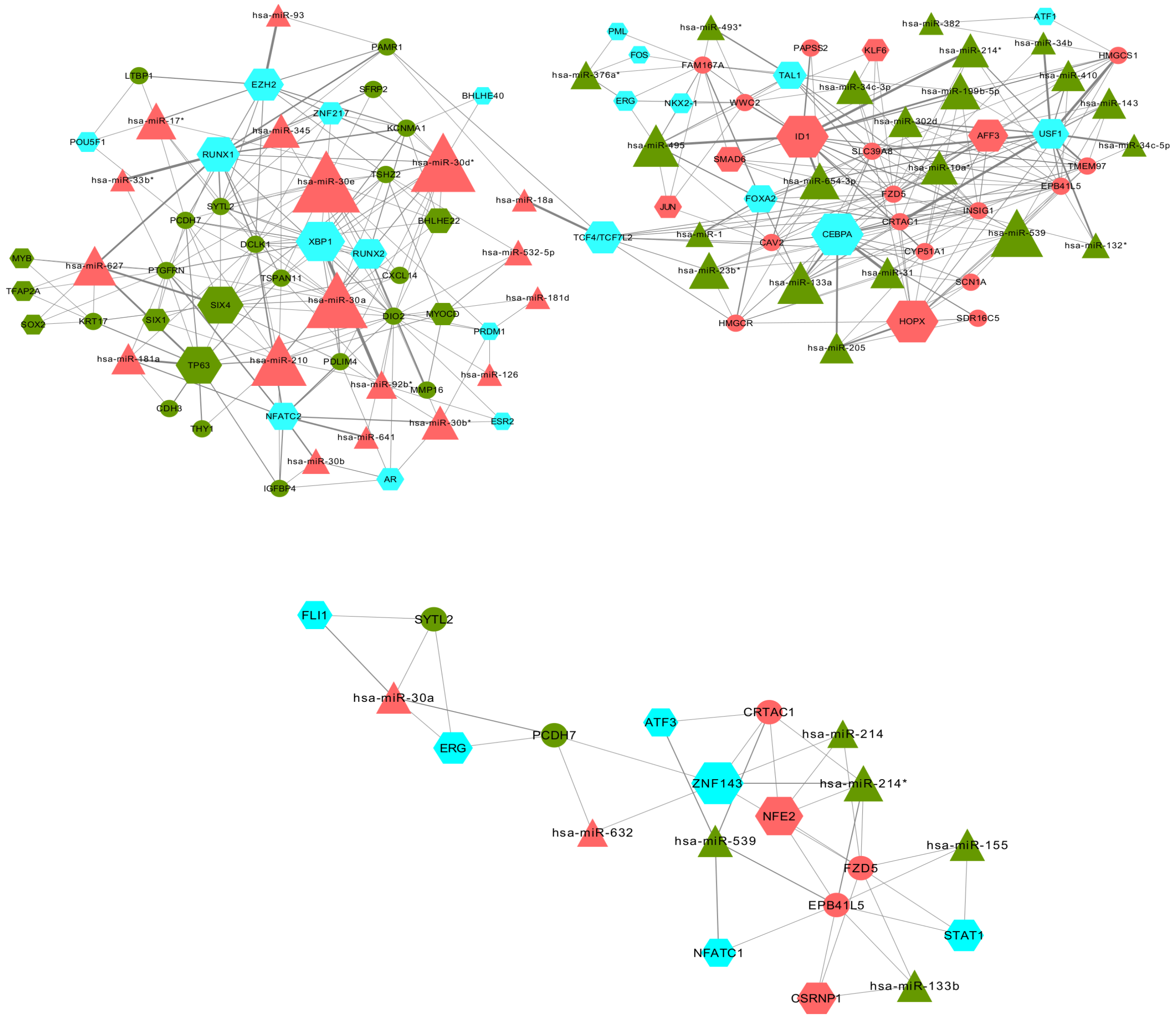

Figure S11: Graphical view of IPF regulatory networks generated for Set A DEGs representing important nodes in the form of IPF genes and miRNAs at each stage (down and up regulated genes and miRNAs are shown in red and green color, respectively). Edges are highlighted based on the edge betweenness and nodes size is adjusted based on outdegree. TFs are shown in sea green color and hexagon shape.
